# Recent Advances in Bioactive Flavonoid Hybrids Linked by 1,2,3-Triazole Ring Obtained by Click Chemistry

**DOI:** 10.3390/molecules27010230

**Published:** 2021-12-30

**Authors:** Daniela Pereira, Madalena Pinto, Marta Correia-da-Silva, Honorina Cidade

**Affiliations:** 1 Laboratory of Organic and Pharmaceutical Chemistry (LQOF), Department of Chemical Sciences, Faculty of Pharmacy, University of Porto, Rua de Jorge Viterbo Ferreira 228, 4050-313 Porto, Portugal; dmpereira@ff.up.pt (D.P.); madalena@ff.up.pt (M.P.); 2 Interdisciplinary Centre of Marine and Environmental Research (CIIMAR), Terminal de Cruzeiros do Porto de Leixões, Av. General Norton de Matos s/n, 4450-208 Matosinhos, Portugal

**Keywords:** flavonoids, Click Chemistry, 1,2,3-triazole, biological activities

## Abstract

As a result of the biological activities of natural flavonoids, several synthetic strategies aiming to obtain analogues with improved potency and/or pharmacokinetic profile have been developed. Since the triazole ring has been associated with several biological activities and metabolic stability, hybridization with a 1,2,3-triazole ring has been increasingly reported over the last years. The feasible synthesis through copper (I) catalyzed azide-alkyne cycloaddition (CuAAC) has allowed the accomplishment of several hybrids. Since 2017, almost 700 flavonoid hybrids conjugated with 1,2,3-triazole, including chalcones, flavones, flavanones and flavonols, among others, with antitumor, antimicrobial, antidiabetic, neuroprotective, anti-inflammatory, antioxidant, and antifouling activity have been reported. This review compiles the biological activities recently described for these hybrids, highlighting the mechanism of action and structure–activity relationship (SAR) studies.

## 1. Introduction

**Figure 1:**
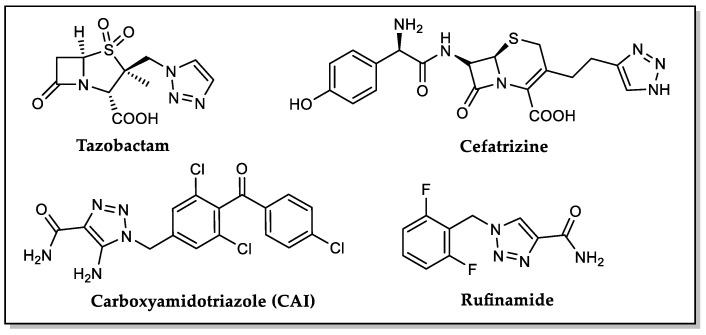
Drugs containing 1,2,3-triazole moiety used in therapeutics.

The 1,2,3-triazole ring in Medicinal Chemistry has been gaining increased attention over the past years. The ability of this heterocycle moiety to participate in hydrogen bonding and dipole interactions provides an improvement of solubility and binding to biomolecular targets. The triazole ring is stable towards hydrolysis, oxidative, and reductive conditions and enzymatic degradation [1]. Moreover, triazole can be used as a linker and as a bioisostere of different functional groups for the synthesis of new active compounds [2,3]. Besides that, therapeutic properties have been attributed to 1,2,3-triazole hybrids, including antibacterial [4], anticancer [5,6,7], and anti-inflammatory [8] activities, among others [3,9]. In fact, some clinically used drugs contain a 1,2,3-triazole moiety, such as the β-lactam antibiotics tazobactam and cefatrizine, as well as the calcium channel blocker carboxyamidotriazole (CAI), and the anticonvulsant rufinamide (Figure 1), reinforcing the importance of this heterocyclic ring in hybridization strategy to obtain new drug candidates with improved efficacy [3,10].

**Figure 2:**
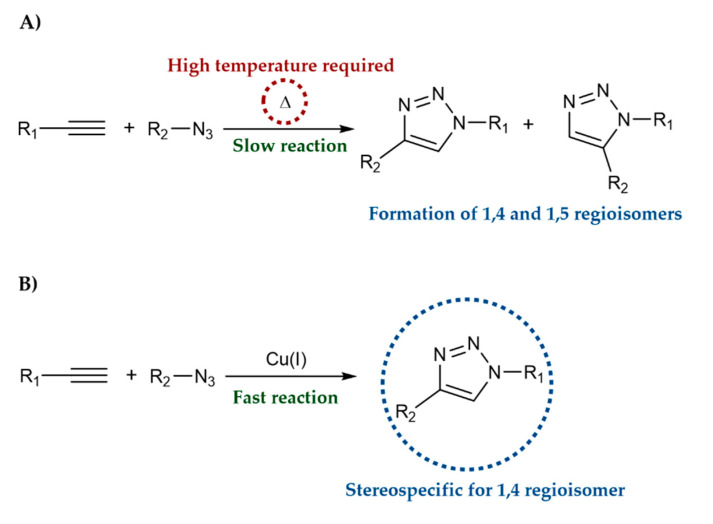
(**A**) Huisgen 1,3-dipolar cycloaddition. (**B**) Copper (I) catalyzed azide-alkyne cycloaddition (CuAAC).

The most common reaction to synthesize the 1,2,3-triazole ring is the copper (I) catalyzed azide-alkyne cycloaddition (CuAAC). CuAAC, also known as Click Chemistry, was developed by the Sharpless and Meldal groups in 2002 and includes the reaction of a terminal alkyne and an aliphatic azide using copper as a catalyst to produce 1,4-disubstituted-triazoles [11,12]. Huisgen 1,3-dipolar cycloaddition (Figure 2A) presented several limitations, including long reaction time, high temperature, and the formation of a mixture of 1,4 and 1,5-disubstitution products. In contrast, CuAAC (Figure 2B) allows for selective, high-yield, faster and mild reaction conditions, with the formation of few side products [13,14].

Moreover, being widely used in drug discovery [15,16], CuAAC has been applied in other areas, including bioimaging [17,18], biological [19], and biomedical [20] applications, synthesis of polymers [21,22] and dendrimers [23,24], and preparation of chemosensors [25], among others.

**Figure 3:**
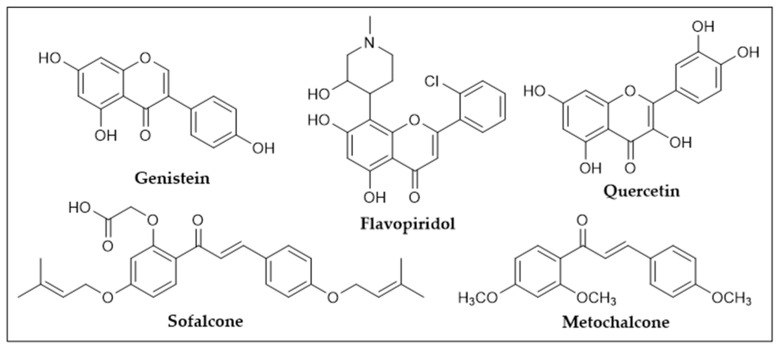
Examples of flavonoids marketed or in clinical trials.

The scaffold associated to flavonoids is very versatile and has long been recognized for the wide range of pharmacological activities, such as anti-inflammatory, cardioprotective, antimicrobial, anticancer, and neuroprotective [26,27,28,29,30,31,32,33,34,35]. The interest in these compounds in Medicinal Chemistry is evidenced by the fact that several flavonoids have been marketed or clinically tested for various health conditions. For instance, quercetin, genistein, and flavopiridol (Figure 3) have been in a late phase of clinical trials for several oncological indications [36] and some chalcones have been marketed or clinically tested, including metochalcone (choleretic/diuretic) and sofalcone (antiulcer/mucoprotective) (Figure 3) [37]. Beyond their potential in Medicinal Chemistry, flavonoids have been reported for their antioxidant, antiaging, anti-browning, anticorrosive, and antifouling activities, which have highlighted the potential of flavonoids for several industrial applications (food, cosmetic, marine industries) [38,39,40,41,42,43].

The hybridization of flavonoids with other pharmacophoric moieties has become a strategy in Medicinal Chemistry for the development of compounds with higher potency by combining two or more pharmacophores into a single entity [44,45]. The incorporation of the 1,2,3-triazole ring in flavonoid hybridization has become a strategy due to its bioactivity, metabolic stability, and convenient synthesis [46,47,48].

**Figure 4:**
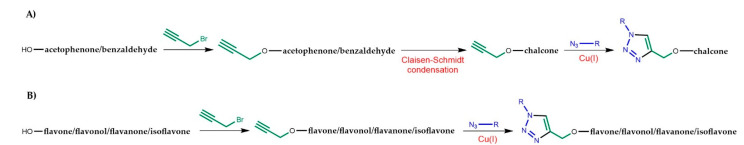
General steps for the synthesis of flavonoid hybrids conjugated with 1,2,3-triazole. (**A**) Synthesis of chalcone-1,2,3-triazole hybrids. (**B**) Synthesis of flavone/flavonol/flavanone/isoflavone-1,2,3-triazole hybrids.

The purpose of this review is to highlight the biological activities of flavonoid hybrids linked by the 1,2,3-triazole ring synthesized in the last five years, emphasizing the mechanism of action and SAR studies. This review covers the articles published from 2017 until November 2021. Scopus, Web of Science, PubMed, and Google Scholar were the databases used for literature retrieval. The methods used for the synthesis of flavonoid hybrids synthesized until 2017 have been exhaustively reviewed by Tomé et al. (2018) [49]. Most of the flavonoid hybrids conjugated with 1,2,3-triazole obtained after 2017 were synthesized by CuAAC with copper sulphate and sodium ascorbate as catalysts, using reaction conditions similar to those previously reviewed by Tomé et al. (2018) [49]. In fact, the in situ generation of Cu(I) salts from copper sulphate through reduction by sodium ascorbate is more convenient and more commonly used than Cu(I) salts, which need the presence of a base as stabilizer and to assist in the ionization of the terminal acetylene [50]. Nevertheless, the utilization of copper iodide (CuI) as a catalyst also continued to be widely reported. Some research papers report the use of other catalysts, such as bromotris (triphenylphosphine) copper (I). Prior to the CuAAC step, a terminal alkyne or an azide is introduced in the flavonoid scaffold. Interestingly, for the synthesis of chalcones, most of the research papers report the propargylation on the building blocks acetophenone or benzaldehyde (Figure 4A), followed by Claisen–Schmidt condensation, whereas for other flavonoids the propargylation generally occurs on the flavonoid scaffold (Figure 4B).

The biological activities of the different subclasses of flavonoid hybrids, synthesized since 2017, are presented as follows, beginning with antitumor activity, for which a higher number of compounds was reported. All new Click Chemistry conditions for this kind of synthesis reported after 2017 will also be highlighted.

## 2. 1,2,3-Triazole-Linked Flavonoid Hybrids with Antitumor Activity

**Figure 5:**
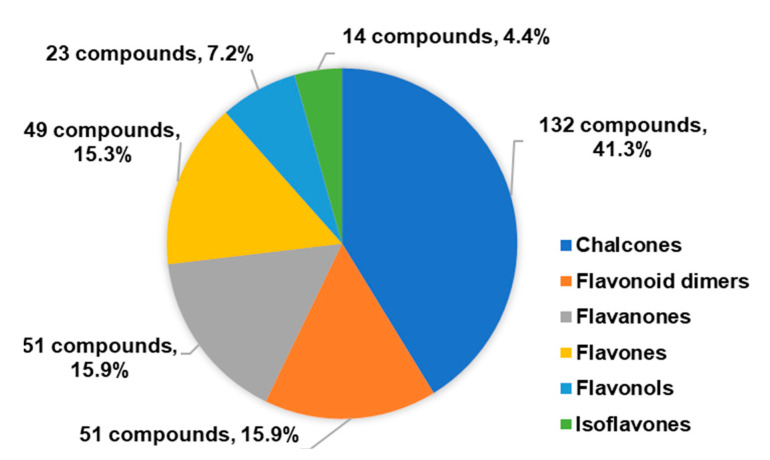
Distribution of the flavonoid hybrids with antitumor activity according to flavonoid subclasses.

More than 300 synthetic 1,2,3-triazole-linked flavonoid hybrids with antitumor activity have been reported since 2017 (Figure 5). Chalcones were the most common flavonoids; however, other classes of flavonoids with antitumor activity were described, namely, flavones, flavanones, flavonols and isoflavones. The conjugation of flavonoids by CuAAC giving rise to bioactive flavonoid dimers was also described.

### 2.1. 1,2,3-Triazole-Linked Chalcone Hybrids

**Figure 6:**
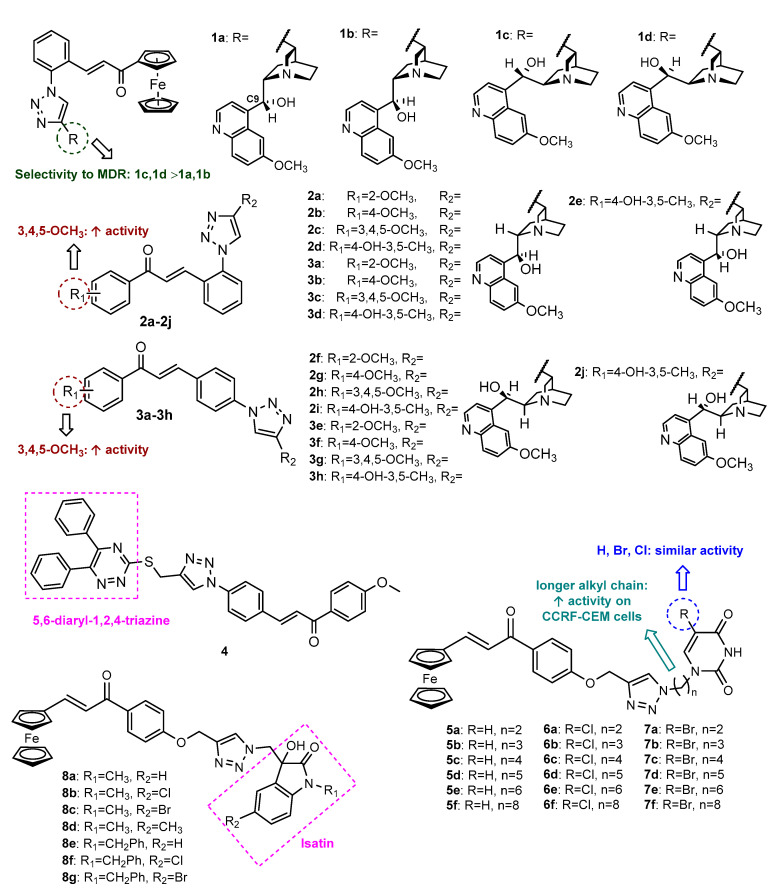
Chalcone hybrids **1**–**8** with antitumor activity.

Considering the application of both ferrocenylchalcones and quinine derivatives in cancer diagnosis and chemotherapy, four ferrocenylchalcones conjugated with quinine/quinidine moieties (**1a**–**1d**, Figure 6) were synthesized by Podolski-Renic et al. (2017) and evaluated for their ability to inhibit the cell growth of three human multidrug-resistant (MDR) cancer cell lines and their sensitive counterparts (non-small cell lung carcinoma NCI-H460/R/NCI-H460, colorectal carcinoma DLD1-TxR/DLD1 and glioblastoma U87-TxR/U87) [51]. The CuAAC reaction between azide ferrocenylchalcones and propargylated quinine/quinidine moieties was performed with specific conditions for the synthesis of different diasterioisomers. The selective synthesis of compounds **1a** and **1c** was performed with 2% copper sulphate and 10% sodium ascorbate, in the presence of neocuproine as a chelating ligand, with 27% and 23% yield, respectively. Otherwise, increasing the proportion of catalyst (copper sulphate to 10% and sodium ascorbate to 50%) without neocuproine led to compounds **1b** and **1d**, with 87% and 72% yield, respectively. All compounds displayed activity against both MDR and non-MDR cancer cells tested, with IC_50_ ranging between 1.60 and 10.71 µM. Among them, compounds **1c** and **1d** were found to display a better selectivity pattern towards MDR cancer cells than epimers **1a** and **1b**. Further studies showed that **1c** and **1d** increased reactive oxygen species (ROS) production and induced mitochondrial damage in MDR cancer cells as well as the inhibition of autophagy. Moreover, simultaneous treatments of **1c** and **1d** with paclitaxel (PTX) increased the sensitivity of MDR cancer cells to PTX [51]. 

Afterwards, the cytotoxic activity of another series of quinine/quinidine-chalcone hybrids linked by a 1,2,3-triazole ring (**2a**–**2j**, **3a**–**3h,** Figure 6) with different substitutions on chalcone A-ring and the triazole ring attached to the *ortho-* (**2a**–**2j**) or *para-* (**3a**–**3h**) position of chalcone B-ring was reported by the same research group [52]. Click Chemistry reaction between the azide chalcone derivatives and propargylated quinine/quinidine moieties provided the final compounds in 21–91% yield. All hybrids showed promising activity against pancreatic PANC-1 (2.43 < IC_50_ < 13.35 µM), colon carcinoma COLO-205 (1.04 < IC_50_ < 7.39 µM), malignant melanoma with high invasiveness A2058 (2.48 < IC_50_ < 18.87 µM) and lung squamous EBC-1 (0.87 < IC_50_ < 7.80 µM) cancer cells, compounds with a 3,4,5-trimethoxybenzene A ring **2c** (IC_50_ = 0.95–2.51 µM), **3c** (IC_50_ = 1.66–2.64 µM), **2h** (IC_50_ = 0.87–6.59 µM) and **3g** (IC_50_ = 1.04–3.72 µM) being the most potent ones.

Trying to obtain new hybrids with antiproliferative activity, 5,6-diaryl-1,2,4-triazine-chalcone hybrid **4** (Figure 6) was synthesized by CuAAC reaction between appropriately substituted azidochalcone and 5,6-diphenyl-3-(prop-2-yn-1-ylthio)-1,2,4-triazine, a heterocyclic skeleton also associated with several biological activities, including antitumor [53]. Compound **4** exhibited moderate antiproliferative activity against gastric cancer cell line MGC-803 (IC_50_ = 36.67 µM), showing no effect over esophageal EC-109 and prostate PC-3 cancer cell lines [53].

Besides antitumor activity, the interest of the application of ferrocene derivatives in the discovery of new therapeutic agents is ascribed for their stability on aqueous and aerobic media, as well as towards air, heat and light, low toxicity, reversible redox, ligand exchange, and catalytic properties [54]. Moreover, uracil derivative 5-fluorouracil (5-FU) is used on the treatment of several cancers. Considering the importance of these moieties, 18 triazole-linked ferrocenylchalcones with uracil (**5a**–**5f**, **6a**–**6f**, **7a**–**7f**, Figure 6) were identified by Singh et al. (2018) as in vitro growth inhibitors of leukemia CCRF-CEM and breast MDA-MB-468 cancer cell lines [55]. Click Chemistry reaction between *O*-propargylated ferrocenylchalcone and *N*-alkylated azido uracil afforded the desired compounds in good yields (75–85%). Among these hybrids, compounds with five, six or eight carbon atom alkyl chains as linkers between uracil and the triazol moiety (**5e**, **5f**, **6d**, **6e**, **7d**, **7e** and **7f**) were shown to be the most potent, reducing the proliferation of CCRF-CEM cancer cells approximately 70% after 72 h, suggesting that the presence of a long alkyl chain is favorable for the activity. Nevertheless, the presence of substituents on the uracil moiety does not seem to have an influence on the activity. Interestingly, further screening against normal kidney LLC-PK1 cell line showed low toxicity after 24 h incubation at 50 µM. Almost all compounds revealed to be inactive against MDA-MB-468, except **5a** and **5d**, which reduced the proliferation by 59% and 62%, respectively [55]. 

Based on the antitumor potential of isatin derivatives, Singh et al. (2018) also reported the synthesis of a series of eight isatin−ferrocenylchalcones linked by a 1,2,3-triazole ring (**8a**–**8g**, Figure 6) and their antiproliferative activity against MDA-MB-231 and MCF-7 breast cancer cells [56]. The *O*-propargylated ferrocenylchalcones were synthesized from propargylated acetophenone and ferrocene-carboxaldehyde, which were after submitted to a CuAAC reaction with *N*-alkylated-azido-isatin derivatives, giving rise to the desired hybrids in 78–85% yield. All compounds showed moderate activity against MCF-7 (IC_50_ = 55.70–70.71 µM), while **8a**, **8c**, **8d**, **8f** and **8g** displayed moderate activity against MDA-MB-231 (IC_50_ = 70.71–96.93 µM).

**Figure 7:**
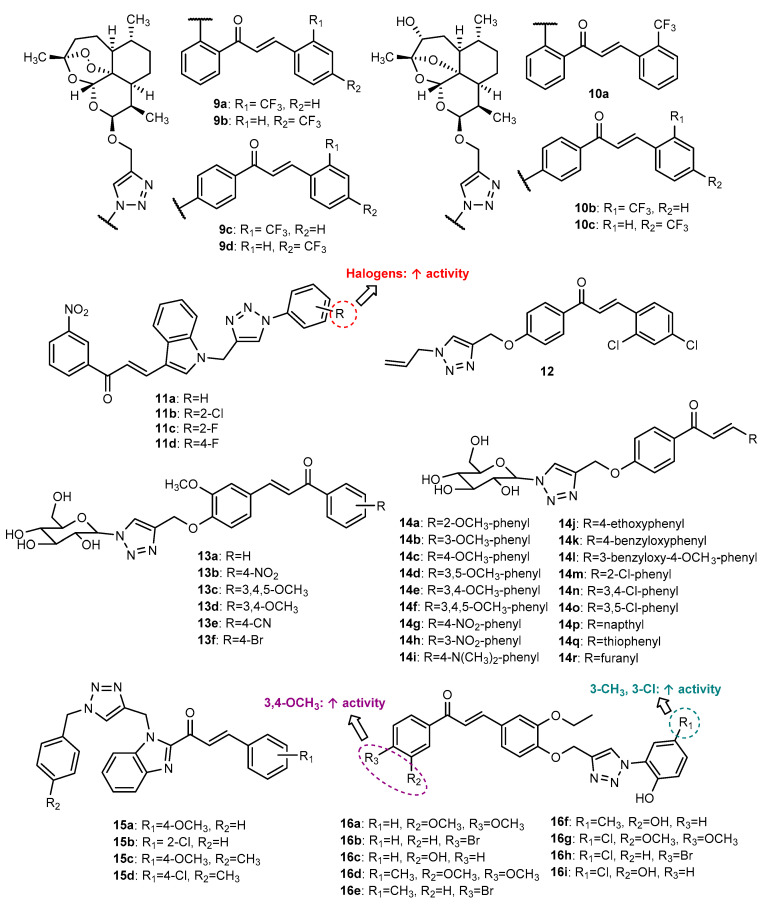
Chalcone hybrids **9**–**16** with antitumor activity.

Kapkoti et al. (2018) reported the antiproliferative activity of seven artemisinin derivatives conjugated with chalcones by a 1,2,3-triazole ring (**9a**–**9d**, **10a**–**10c**, Figure 7), synthesized through the Click Chemistry approach [57]. After the *O*-propargylation of artemisinin derivatives, the CuAAC reaction occurred with different substituted azide chalcones. These compounds were screened against six human cancer cell lines, namely, erythromyeloblastoid leukemia (K562 cell line), squamous (PC-3 and A431), breast (MDAMB-231), and lung (COLO-205 and A549) carcinoma, as well as against the normal kidney cell line HEK-293 and showed moderate-to-good antiproliferative activity against at least one cancer cell line (IC_50_ = 7.16–69.67 µM), with no toxicity for normal cells. Compound **9a** displayed the highest activity against all cancer cells tested (IC_50_ = 7.16–28.21 µM), this effect being associated with G2/M cell cycle arrest, ROS formation and apoptosis in A431 and A549 cell lines. Additionally, the toxicity study on human erythrocytes revealed that **9a** was non-toxic even at the highest tested concentration (100 µg/mL).

Considering that indole derivatives, namely, indole-chalcones, have been reported due their antitumor potential, and aiming to combine this scaffold with a 1,2,3-triazole ring, four *N*-substituted 1,2,3-triazolyl-linked indole-chalcone hybrids (**11a**–**11d**, Figure 7) were synthesized by Aneja et al. (2018) and screened for their in vitro antitumor activity against cervical SiHa and SW620 cancer cell lines [58]. The *N*-propargylation of indole-chalcone, followed by CuAAC reaction with substituted aryl azides at room temperature, afforded the final compounds in good yields (68–84%). Hybrids **11b** (IC_50_ = 48.96–67.99 µg/mL), **11c** (IC_50_ = 63.22–76.71 µg/mL) and **11d** (IC_50_ = 99.30–103.09 µg/mL) displayed higher activity than **11a** (IC_50_ = 144.68–171.89 µg/mL) in cancer cell lines and showed low cytotoxic effect in normal embryonic kidney cells HEK293, suggesting that the presence of halogens in the benzene ring is associated with an increased activity. Moreover, DNA binding and docking studies showed that compounds **11b**–**11d** bind to DNA via the non-covalent intercalative mode causing the antiproliferative activity, hybrid **11b** being identified as the hit compound [58].

The novel triazole-chalcone hybrid **12** (Figure 7) was synthesized by Yan et al. (2019) and evaluated for its in vitro growth inhibitory activity against liver cancer cell lines [59]. The *O*-propargylation of 2,4-dichloro-4′-hydroxychalcone and the CuAAC reaction occurred in the same step, in the presence of 3-azidoprop-1-ene, propargyl bromide, copper sulphate and sodium ascorbate in THF/water, at room temperature, giving rise to the desired compound in good yield (86%). Hybrid **12** displayed a potent antiproliferative effect against all cancer cell lines tested (IC_50_ = 0.9 μM, 2.7 μM, 6.2 μM and 4.6 μM for HepG2, SNU-423, SMMC7221, and SNU-398, respectively), higher than the positive control 5-fluorouracil (5-FU) (IC_50_ = 9.2–17.9 μM) and 2,4-dichloro-4′-hydroxychalcone (IC_50_ values > 20 μM), showing that the 1,2,3-triazole unit has an important role in the antiproliferative activity. Since hybrid **12** exhibited the highest activity against HepG2, this cell line was chosen for further studies. It was found that **12** inhibits HepG2 cell growth and colony formation in a concentration-dependent manner. Moreover, compound **12** inhibits liver cancer cell migration by regulating epithelial–mesenchymal transition (EMT)-related biomarkers (E-cadherin, N-cadherin and vimentin) and inhibiting the expression of upstream transcription factors (Snail and Slug). Additionally, this compound showed a potent tubulin polymerization inhibitory effect (IC_50_ = 2.34 μM). Further studies showed that **12** inhibited the liver cancer cell growth in mice, with low toxicity [59].

Manna et al. (2019) reported the synthesis of two series of glycosyl-chalcone derivatives linked by the 1,2,3-triazole, namely, hybrids **13a**–**13f** with different substitutions on chalcone A ring and hybrids **14a**–**14r** with different substitutions on chalcone B ring (Figure 7) [60]. The synthesis was performed in several steps, starting with the *O*-propargylation of benzaldehyde or acetophenone, which were used after as building blocks for the synthesis of propargylated chalcones by Claisen–Schmidt condensation. Click Chemistry reaction with glycosylated azide gave rise to the acetylated conjugates with 68–77% yield. Then, the de-*O*-acetylation of glycosyl moiety afforded final compounds **13a**–**13f** and **14a**–**14r** in quantitative yield. All compounds showed cytotoxic potential against MDA-MB-468 (LD_50_ = 28–89 µM) and MCF-7 (LD_50_ = 31–123 µM) cancer cell lines. Compounds were also screened against a non-cancerous lung fibroblast cell WI-38. Hybrid **14i** was identified as the most promising compound (LD_50_ = 28 µM, 31 µM and 59 µM for MDA-MB-468, MCF-7 and WI-38, respectively), showing the highest selectivity between normal and cancer cells, with a selectivity index of 2.11 (WI-38/ MDA-MB-468) and 1.9 (WI-38/ MCF-7). Further studies showed that the treatment of MDA-MB-468, MCF-7 and WI-38 cells with **14i** promoted apoptosis in both MDA-MB-468 and MCF-7 cells in a concentration dependent manner, while for WI-38 non-cancer cell there was no substantial apoptosis. Moreover, compound **14i** induced oxidative damage through the production of ROS, and induced cell cycle arrest, DNA fragmentation and apoptosis in MDA-MB-468 and MCF-7 cancer cells, without affecting the WI-38 non-cancerous cells. The Western blot analysis of MDA-MB-468 cells treated with **14i** showed a significant change in Bcl-2-associated X protein (Bax)/B-cell lymphoma 2 (Bcl-2) ratio and activation of caspase-3, which play a critical role in cancer cell apoptosis [60].

In an attempt to develop new heterocyclic hybrids, and considering the biological properties ascribed to the benzimidazole nucleus, including antitumor, Djemoui et al. (2020) reported the synthesis and cytotoxic potential of four benzimidazole-chalcone hybrids conjugated with 1,2,3-triazole (**15a**–**15d**, Figure 7) [61]. The *N*-propargylation of benzimidazole-chalcone intermediate, followed by Click Chemistry reaction with respective benzyl azides, gave rise to the final compounds in good-to-excellent yield (81–91%). Hybrid **15b** showed potent activity against breast cancer cells T47-D and MDAMB-231 and the prostate PC3 cancer cell line (IC_50_ of 6.23, 5.89 and 10.7 µM against T47-D, MDA-MB-231 and PC3, respectively), and compound **15d** showed the highest activity in the PC3 cell line (IC_50_ = 5.64 µM), with selectivity for this cancer cell line, whereas compounds **15a** and **15c** showed moderate activity against all tested cancer cells (IC_50_ = 36.7–87.6 µM). 

The synthesis of chalcone hybrids **16a**–**16i** (Figure 7) was achieved in three steps, starting with the *O*-propargylation of benzaldehydes, which were further submitted to the CuAAC reaction with phenyl azides, giving the intermediate benzaldehydes in good yields (75–83%). The Claisen–Schmidt condensation of these benzaldehydes with substituted acetophenones afforded the desired compounds in 68–87% yield [62]. All compounds were screened for their in vitro cytotoxic activity against MCF-7, HeLa and MDA-MB-231 cancer cell lines. While MCF-7 was found to be more sensitive (IC_50_ = 0.02–4.15 µM after 48 h incubation), HeLa cells were found to be more resistant (IC_50_ = 31.60–177.7 µM after 48 h incubation) for all tested compounds. For MDA-MB-231 cancer cells, IC_50_ values ranged between 0.31 and 60.92 µM. Hybrids **16d** (IC_50_ = 0.78–31.60 µM), **16g** (IC_50_ = 0.02–71.05 µM) and **16i** (IC_50_ = 1.59–31.60 µM) were found as the most promising cytotoxic compounds, showing low toxicity against the non-cancerous cells MCF-10a. Specifically, **16g** displayed higher cytotoxicity for MCF-7 and MDA-MD-231 (IC_50_ = 0.02 and 0.31 µM) than the anticancer drug *cis*-platin (IC_50_ = 1.28 and 7.34 µM, respectively). Molecular docking studies identified **16i** with the highest dock score for EGFR kinase, whereas **16c** obtained the highest dock score for estrogen receptor α (ERα). ADMET in silico studies showed satisfactory oral bioavailability for all compounds. The presence of 3,4-dimethoxybenzene A ring on the chalcone scaffold, as well as the presence of *meta*-substituted chloro and methyl groups on the benzene ring, is associated with an increase in activity when compared with other derivatives [62].

**Figure 8:**
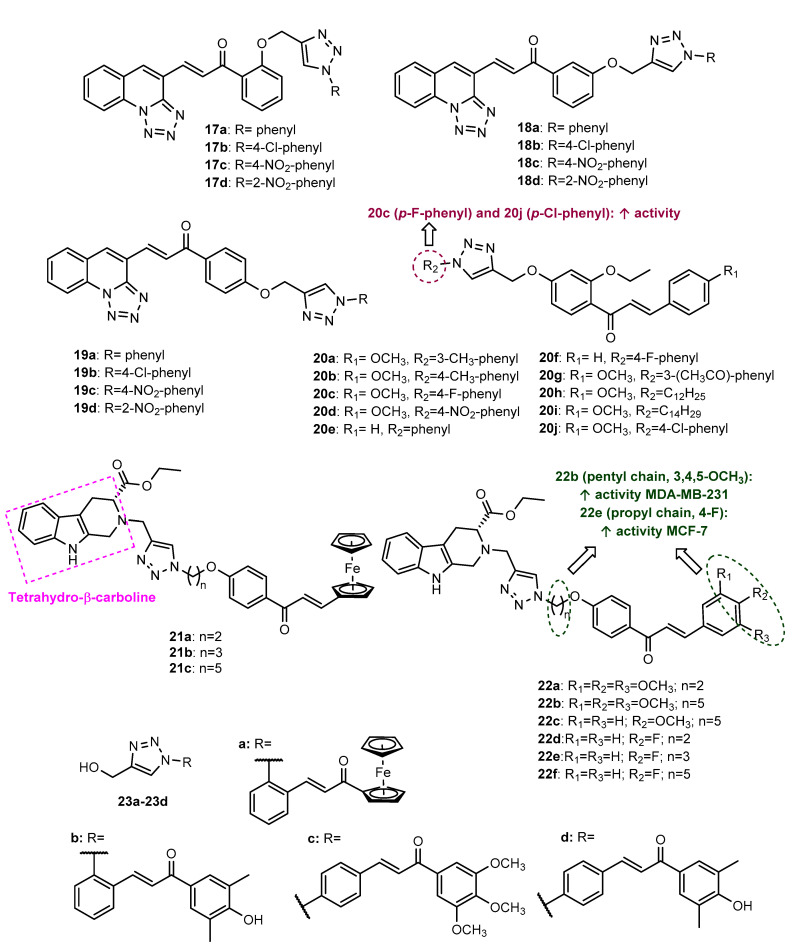
Chalcone hybrids **17**–**23** with antitumor activity.

Nagaraju et al. (2020) reported the synthesis and antiproliferative activity of 12 tetrazolo-quinoline-chalcones conjugated with triazole ring (**17a**–**17d**, **18a**–**18d** and **19a**–**19d**, Figure 8) via Click Chemistry reaction from *O*-propargylated chalcones and aryl azides [63]. All the compounds were found to display potent antitumor activity against three cancer cell lines, SiHa, MDA-MB-231 and PANC-1 (GI_50_ = 0.44–2.67), higher than the positive control doxorubicin (DOX) (GI_50_ = 1.15–3.10).

Ten 1,2,3-triazole-linked chalcone hybrids (**20a**–**20j**, Figure 8) were synthesized by CuAAC reaction from *O*-propargylated chalcones and aryl or alkyl azides [64]. All compounds were shown to have moderate-to-low activity against the A549 cancer cell line (IC_50_ = 62.51–174.27 µM), compared to the positive control DOX (IC_50_ = 39.86 µM). Among them, compounds **20c** (IC_50_ = 62.51 µM) and **20j** (IC_50_ = 75.41 µM), having *para*-fluoro and *para*-chloro groups, allied to the presence of the methoxy group on the B ring of the chalcone scaffold, showed the highest activity. 

Considering the antitumor potential of the natural alkaloid tetrahydro-β-carboline and the role of synthetic tetrahydro-β-carboline derivatives against breast cancer cells, Sharma et al. (2020) prepared a series of tetrahydro-β-carboline-chalcone/ferrocenylchalcone hybrids linked by a 1,2,3-triazole ring (**21a**–**21c** and **22a**–**22f**, Figure 8), which were further screened for their antiproliferative activity against estrogen-responsive MCF-7 and triple negative MDA-MB-231 cell lines [65]. The Click Chemistry reaction was achieved using *N*-propargylated tetrahydro-β-carboline and *O*-alkylazido-ferrocenylchalcones or *O*-alkylazidochalcones as precursors. Among the synthesized compounds, ferrocenylchalcones **21a**–**21c** were shown to be selective to the MCF-7 cell line with moderate activity (IC_50_ = 70.71–79.3 µM). Chalcones with an aryl B ring, **22a**, **22d** and **22f,** also showed selectivity for MCF-7 cells (IC_50_ = 19–68.61 µM), whereas **22b** and **22e** displayed moderate activity against both cancer cells (IC_50_ = 44.73 and 10.33 µM for MCF-7 and IC_50_ = 21.99 and 70.71 µM for MDA-MB-231, respectively). Compound **22c** showed selectivity for MDA-MB-231 cancer cells, with an IC_50_ of 70.71 µM. Compound **22e**, with a 4-fluoro substituent and a propyl chain as spacer, showed the highest activity for MCF-7 cells (IC_50_ = 10.33 µM), while **22b**, bearing a 3,4,5-trimethoxybenzene B ring and a pentyl chain as a spacer, showed the highest activity for MDA-MB-231 cells, being five- and three-fold more potent than the positive control tamoxifen, respectively.

Compounds **23a**–**23d** (Figure 8), reported by Latif et al. (2020), were screened for their cytotoxic activity against the estrogen receptor (ER)-positive breast cancer cell line MCF-7 and the triple-negative breast cancer (TNBC) MDA-MB-231 cell line [66]. Although the activity of ferrocenylchalcone **23a** was lower than 10% at 20 µM for both cancer cells, **23b**–**23d** showed considerable cytotoxic activity for MCF-7 (2.51 < IC_50_ < 15.07 µM) and MDA-MB-231 cells (4.40 < IC_50_ < 11.11 µM), **23b** being the most active for both cells, better than the positive control cisplatin.

### 2.2. 1,2,3-Triazole-Linked Flavonoid Dimers

**Figure 9:**
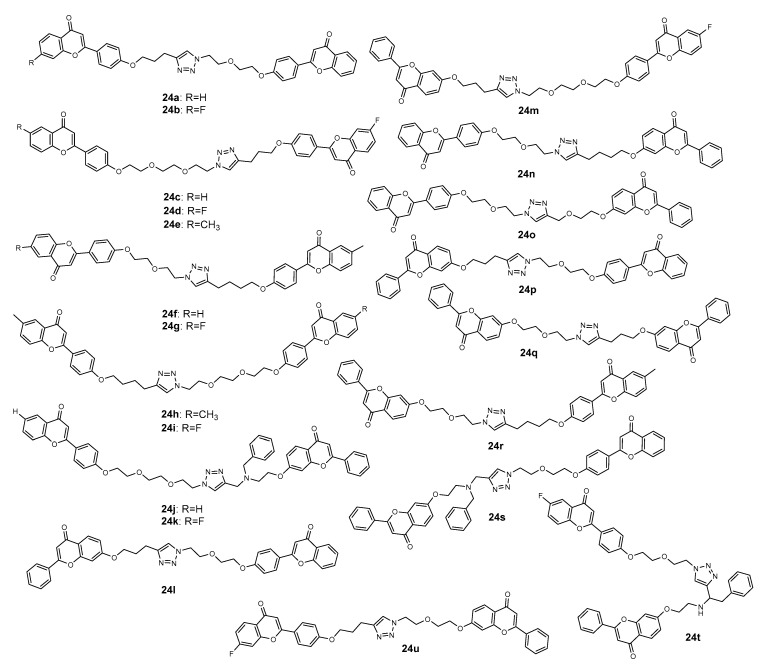
Flavonoid dimers **24a**–**24u** as promising MRP1 inhibitors.

Wong et al. (2018) reported the synthesis of a combinatorial library of 300-member flavonoid dimers linked by triazole and the screening of the effect of these dimers on the inhibition of multidrug-resistance-associated protein 1 (MRP1, ABCC1) [67]. Authors referred the strategy of combining two flavonoid moieties due to the pseudodimeric structure of many ATP-binding cassette (ABC) transporters, which includes P-gp/ABCB1, MRP1/ABCC1, and BCRP/ABCG2, the three major ABC members that confer cancer MDR. The high-throughput screening of this library allowed us to identify 21 dimers (**24a**–**24u**, Figure 9) with a high level of activity and safety profile. These hybrids were further synthesized on a macroscale, and complementary assays were carried out. First, a library of propargylated and azide flavonoid derivatives were prepared. Then, CuAAC reactions were conducted in the presence of bromotris (triphenylphosphine) copper (I) as catalyst in THF, under reflux, to afford the final compounds with moderate-to-excellent yields (24–92%). All these dimers displayed a remarkable MRP1 modulatory effect (53 < EC_50_ < 298 nM), higher than the well-known MRP1 inhibitors verapamil (EC_50_ = 1950 nM) and MK571 (EC_50_ = 19,000 nM). Moreover, these compounds were relatively nontoxic toward L929 cells (selective indexes ranged from >190 to >1887). Among them, **24u** was the most potent (EC_50_ = 53 nM) and safe (selective index > 1887), and it was predicted to bind to the bipartite substrate-binding site of MRP1 in a competitive manner. Moreover, it provided a concentration sufficient for maintaining the plasma level above its in vitro EC_50_ (53 nM for DOX) for about 90 min in Balb/c mice [67]. 

**Figure 10:**
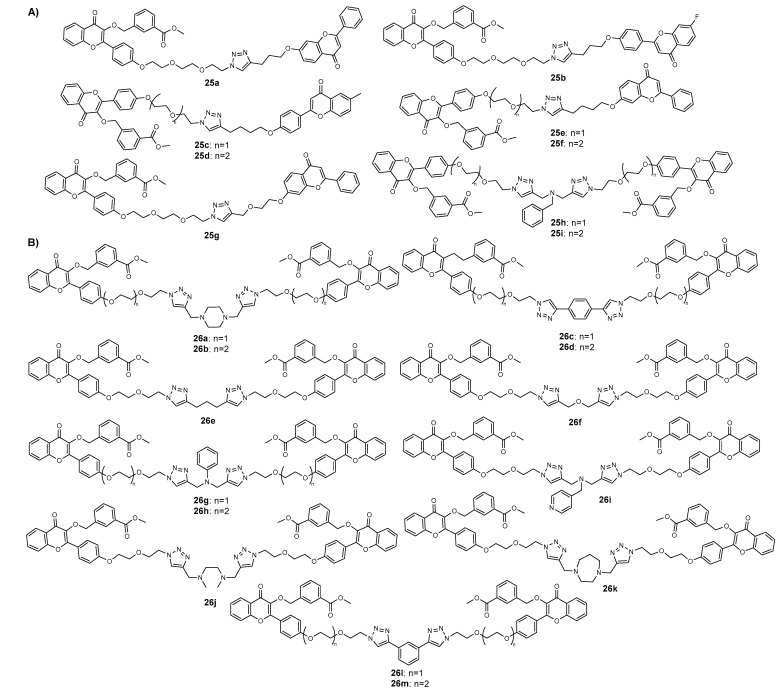
(**A**) First series of flavonoid dimers **25a**–**25i** reported by Zhu et al. [68]. (**B**) Second series of flavonoid dimers **26a**–**26m** with BCRP inhibitory effect reported by Zhu et al. [68].

Afterwards, in order to find new selective breast cancer resistant protein (BCRP) inhibitors, the same research group reported the synthesis of a series of 74 homo- and hetero-flavonoid dimers with mono- or bis-triazole ring and the evaluation of their potential to inhibit BCRP, MRP1, and P-glycoprotein (P-gp) transporter proteins [68]. The CuAAC step using propargylated and azide flavonoids in the presence of bromotris (triphenylphosphine) copper (I) in THF, under reflux, allowed us to obtain dimers bearing one triazole ring with 24–98% yield. Compounds having a bis-triazole ring were produced in 49–99% yield by conjugating azide flavonoids with diacetylenes. Among these flavonoid dimers, compounds **25a**–**25i** (Figure 10A) and **24d**, **24u**, reported previously (Figure 9) [67], exhibited potent MDR inhibitory activity against BCRP for the two cell lines HEK293/R2 and MCF7-MX100 in restoring topotecan cytotoxicity, showing compounds **25h** and **25i** a higher activity for BCRP than MRP1 and P-gp [68]. Using **25h**–**25i** as models, a series of 13 analogues (**26a**–**26m**, Figure 10B) were then synthesized, the bis-triazole bridged flavonoid dimer **26l** being identified as the most potent BCRP inhibitor (EC_50_ = 1−2 nM), with high selectivity compared to MRP1 and P-gp proteins [68]. Moreover, this compound was found to inhibit BCRP-ATPase activity and block the efflux activity of BCRP, thus restoring the drug sensitivity in BCRP-overexpressing cells. Therefore, dimers **26a**–**26m** appear to be promising candidates for further development into combination therapy to overcome MDR cancers with BCRP overexpression [68].

**Figure 11:**
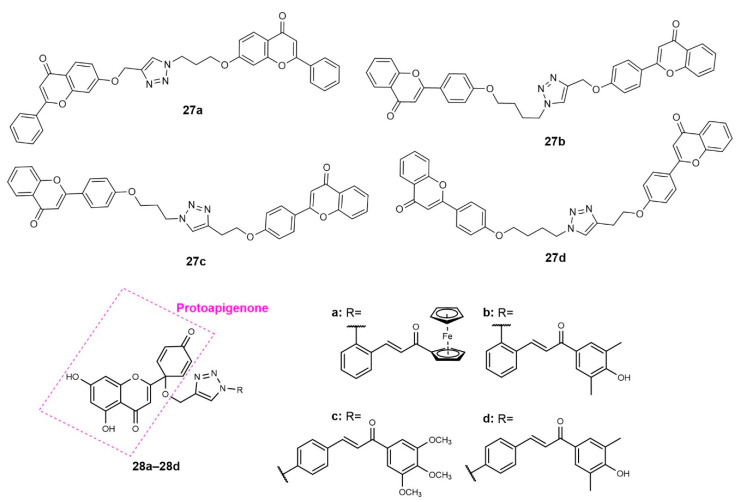
Flavonoid dimers **27**–**28** with antitumor activity.

In another study, flavonoid dimers **27a**–**27d** (Figure 11) showed moderate antiproliferative activity on different cell lines, including colon HCT116, HepG2, MCF-7 and leukaemia MOLT-4 [69]. The CuAAC reaction step was performed between propargylated and halide flavones in the presence of copper sulphate, copper turnings, sodium azide and sodium ascorbate in a mixture of *t*-butanol/water. Using these reaction conditions, the azide was formed in situ during Click Chemistry reaction. Interestingly, authors reported this reaction at room temperature, under reflux and microwave (MW) irradiation, and showed that the last method reduced the reaction time from 48–96 h to just 0.25 h, with 63–72% yield. Dimers **27a**, **27b** and **27d** displayed low-to-moderate cell growth inhibition against some of the tested tumor cell lines (27.25 < IC_50_ < 68.60 µM), with no cytotoxicity against the immortalized keratinocyte HaCaT non-tumor cell line. Nevertheless, compound **27c,** which showed an in vitro growth inhibitory effect on MOLT-4 (IC_50_ = 24.3 µM), also exhibited growth inhibitory activity on HaCaT non-tumor cell (IC_50_ = 47.42 µM) [69].

Based on the ability of a protoflavone, the protoapigenone, to inhibit the ataxia-telangiectasia and Rad3-related protein (ATR) and to induce the oxidative stress and the pro-oxidant activity of chalcones, Latif et al. (2020) prepared a series of heterodimers **28a**–**28d**, containing a protoflavone and a chalcone residue (Figure 11), in order to combine these two pharmacological activities [66]. The Click Chemistry step was performed from *O*-propargylated protoapigenone and chalcone azides. The obtained compounds were screened against the estrogen receptor (ER)-positive breast cancer cell line MCF-7 and the triple-negative breast cancer (TNBC) MDA-MB-231 cell line. All hybrids were shown to have a potent activity against both cancer cells (0.25 < IC_50_ < 0.51 µM and 0.22 < IC_50_ < 0.37 µM for MCF-7 and MDA-MB-231, respectively), higher than the positive control cisplatin (IC_50_ = 5.35 and 26.15 µM). The activity was compared with chalcone triazole conjugates **23a**–**23d** (Figure 8), reported in Section 2.1, to evaluate the influence of hybridization. It was found that hybrids **28a**–**28d** presented a higher activity than propargylated protoapigenone (IC_50_ = 1.74 and 2.53 µM) and compounds **23a**–**23d**, which confirmed the synergic effect of the hybridization. Moreover, whereas hybrids **28a**–**28d** showed an induction of late apoptosis and necrosis on MCF-7 cells, only hybrids **28c** and **28d** decreased the viability of MDA-MB-231 cells. Compound **28c** also increased the hypodiploid (subG1) phase on MDA-MB-231 cells, which indicate apoptotic cell death and inhibition of the DNA synthesis in a dose-dependent manner. This hybrid also caused a time- and concentration-dependent increase in caspase-3 activity. Hybrids **28a**–**28d** also displayed a potent inhibition of the ATR-dependent activation of Chk1, which can explain their strong cytotoxicity on the TNBC cell line MDA-MB-231. The effects of **28a**–**28d** on intracellular ROS and/or reactive nitrogen species (RNS) levels were studied, and all the compounds displayed ability to interfere with the redox system in breast cancer cells. Moreover, all compounds induced significant depolarization of the mitochondrial membrane in both cell lines [66].

### 2.3. 1,2,3-Triazole-Linked Flavanone Hybrids

**Figure 12:**
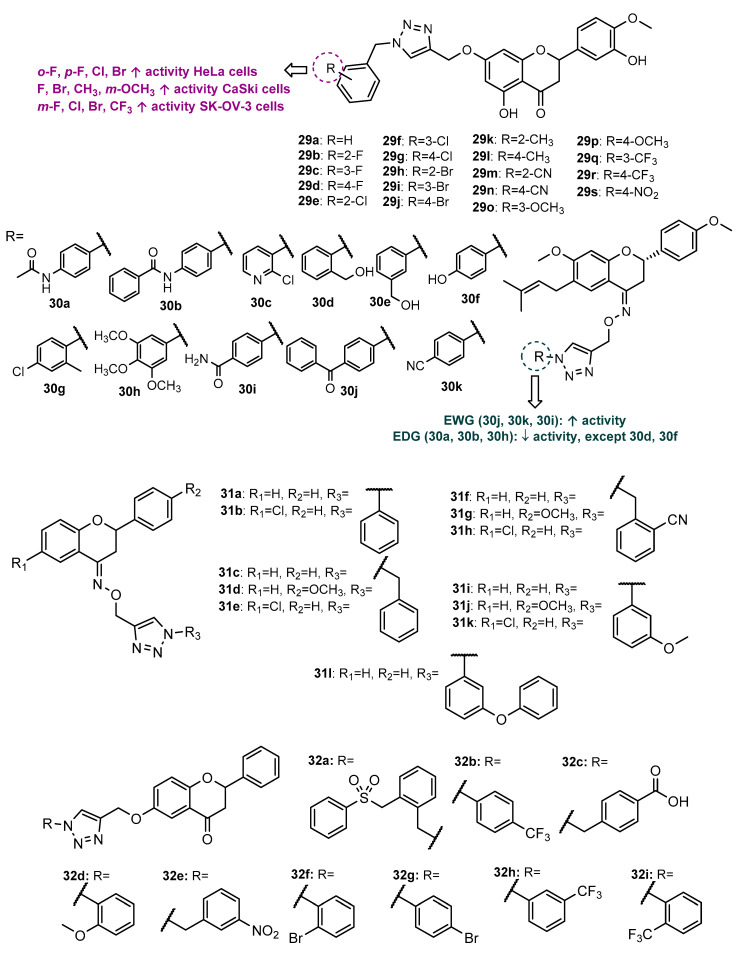
Flavanone hybrids **29**–**32** with antitumor activity.

A series of hesperetin hybrids linked by the 1,2,3-triazole ring (**29a**–**29s**, Figure 12) were synthesized by molecular modification of hesperitin-7-rhamnoglucoside and screened for antiproliferative activity against HeLa, cervical CaSki and SK-OV-3 cancer cells [70]. After the *O*-propargylation of hesperitin, CuAAC reaction with benzyl azides afforded **29a**–**29s** with 40–85% yield. All compounds displayed moderate antiproliferative activity against HeLa (17.8 < IC_50_ < 102.8 µM), CaSki (12.7 < IC_50_ < 77.8 µM) and SK-OV-3 (33.3 < IC_50_ < 95.3 µM) cancer cells, with bearable toxicity for the Madin–Darby canine kidney (MDCK) non-cancer cell line (179.8 < CC_50_ < 334.7 µM). Overall, the results suggest that the presence of EWG on the benzyl ring attached to 1,2,3-triazole moiety is associated with an improvement of the antiproliferative activity against all cancer cells (Figure 12).

Gupta et al. (2018) reported the synthesis and antiproliferative activity against A549, PC-3, HCT-116 (Human colon carcinoma) and MCF-7 cancer cells of 1,2,3-triazole-linked bavachinin derivatives **30a**–**30k** (Figure 12), obtained by molecular modification of the natural flavanone bavachinin [71]. The modification of bavachinin scaffold, followed by *O*-propargylation and Click Chemistry reaction with different substituted azides, gave rise to the final compounds in excellent yields (85–95%). First, screening of all compounds at 50 µM against the four cancer cells identified derivatives **30d** and **30i** as the most promising compounds with 100% growth inhibition. **30i** was found to be the most potent with IC_50_ values of 7.72, 16.08, 7.13 and 11.67 µM against A549, PC-3, colon HCT-116 and MCF-7, respectively. When comparing the growth inhibitory activity of **30i** with bavachinin, it was verified that this derivative exhibited three- and four-fold improvement in cytotoxicity against HCT-116 and A549 cell lines. Mechanistic studies showed that **30i** inhibited colony formation and in vitro migration of HCT-116 cells. Moreover, it induced morphological changes and mediated the apoptotic cell death of HCT-116 cells with perturbance in MMP and PARP cleavage [71]. Overall, analogues bearing EWG showed higher activity than analogues with electron-donating groups (EDG), except for **30d**, having the -CH_2_OH group at *o-*position and **30f**, with a -OH group at *p*-position (Figure 12).

A series of 12 flavanone hybrids **31a**–**31l** (Figure 12), synthesized by CuAAC from *O*-propargylated flavanone derivative and different substituted azides, showed some cytotoxic activity for at least one of the cancer cells tested (HCT-15, HeLa and NCI-H522), with IC_50_ values ranging from 5.4 to 46.6 µM [72]. All compounds, except **31a**, were shown to have low toxicity for normal cell HEK-293. Among them, **31b** showed the highest activity for NCI-H522 (IC_50_ = 5.4 µM), while **31d** displayed good antiproliferative activity against all tested cancer cells (15.6 < IC_50_ < 17.4 µM).

BCR-ABL kinase inhibitors are usually the first line for the treatment of Chronic Myeloid Leukaemia. In order to discover new BCR-ABL kinase inhibitors, Ribeiro et al. (2021) synthesized nine flavanone hybrids (**32a**–**32i**, Figure 12) [73]. After the propargylation of 6-hydroxyflavanone, the Click Chemistry step with different substituted azides was performed under MW irradiation, in the presence of copper sulphate and sodium ascorbate in DMF, for 10 min. Although most of the synthesized compounds showed a slight inhibition of BCR-ABL kinase, **32c** and **32d** showed an activity on a nanomolar scale, with IC_50_ values of 0.364 and 0.275 µM, respectively. Docking studies and molecular dynamic simulations performed for the most active compound, **32d**, in the kinase domain of BCR-ABLWT revealed favorable interactions of this hybrid with important amino acid residues, similar to imatinib, in the ATP-binding pocket site, and **32d** induced conformational changes in the structure of BCR-ABL. In silico pharmacokinetic predictions showed that the two most active compounds, **32c** and **32d**, had a profile similar to imatinib. Compound **32d** was also shown to passively permeate through the blood–brain barrier (BBB), while imatinib and **32c** only were predicted to pass through the central nervous system by P-glycoprotein. Moreover, in silico toxicity evaluation showed that **32c** had less toxicity potential than imatinib in six of ten toxicity parameters tested [73].

### 2.4. 1,2,3-Triazole-Linked Flavone Hybrids

**Figure 13:**
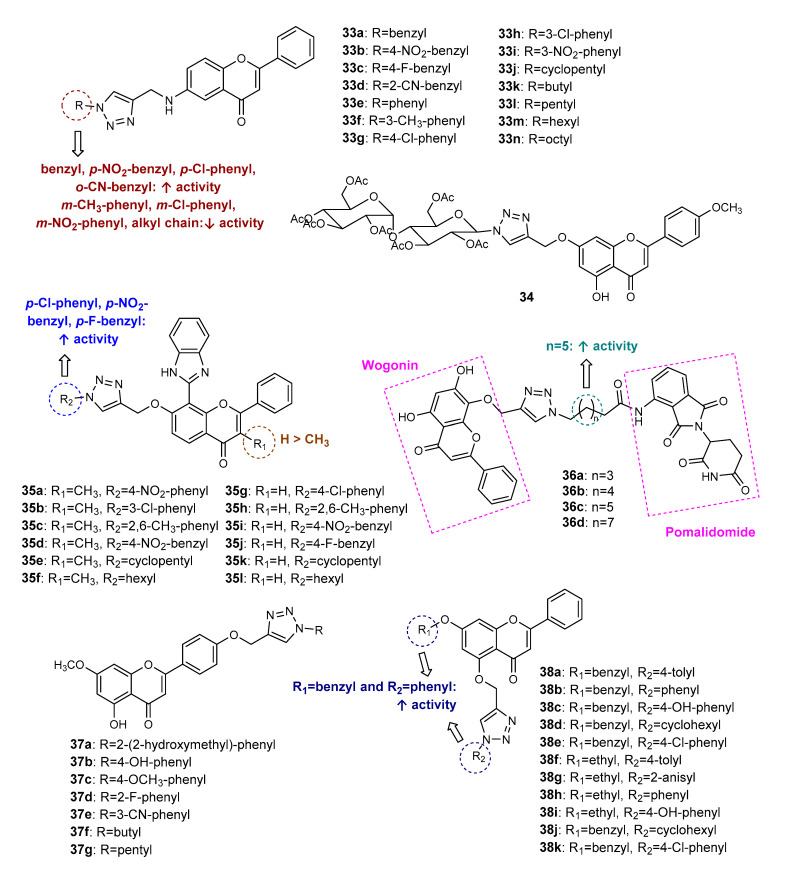
Flavone hybrids **33**–**38** with antitumor activity.

Flavone hybrids **33a**–**33n** (Figure 13) were synthesized by Sowjanya et al. (2017) and screened for their in vitro antiproliferative activity against four human cancer cell lines (HeLa, pancreatic MIA PaCa, MDA-MB-231 and neuroblastoma IMR 32) [74]. The *N*-propargylation of previously synthesized flavone, followed by CuAAC reaction with alkyl or aryl azides, afforded the desired compounds with 61–82% yield. All hybrids displayed moderate-to-potent antiproliferative activity against MIA PaCa, MDA-MB-23 and IMR 32 (0.01 < GI_50_ < 34.0 µM), while for HeLa the tested compounds were shown to have moderate-to-low activity (25.8 < GI_50_ < 100 µM). Among them, **33b** displayed the highest activity against MDA-MB-231 (GI_50_ < 0.01 µM), better than reference drugs DOX (GI_50_ = 0.085 µM) and PTX (GI_50_ = 0.091 µM). Compounds **33a**, **33d** and **33g** also exhibited promising antiproliferative activity against MIA PaCa, MDA-MB-231 and IMR 32 (0.11 < GI_50_ < 1.5 µM). Hybrids **33b**, **33c**, **33e** and **33i** showed moderate activity against HeLa cells (25.8 < GI_50_ < 29.2 µM). The SAR suggests that the presence of electron-withdrawing groups (EWG) on the benzene ring attached to the 1,2,3-triazole moiety increases the antiproliferative activity, as illustrated in Figure 13. On the contrary, when comparing the GI_50_ values of all compounds, we suggest that the presence of an alkyl chain or the substituents at *meta* position on the benzene ring linked to the 1,2,3-triazole decreases antiproliferative activity.

Wang et al. (2018) reported the synthesis and antitumor activity of 1,2,3-triazole-linked flavone-glycoside **34** (Figure 13) [75]. After the *O*-propargylation of flavone derivative, CuAAC reaction was accomplished with acetyl glycosyl azide, giving rise to **34** with 68% yield. Compound **34** showed promising activity against HeLa cells (IC_50_ = 14.67 µM), higher than the positive control *cis*-platin (IC_50_ = 21.30 µM).

The antiproliferative activity of 12 flavone hybrids (**35a**–**35l**, Figure 13) against MCF-7 cancer cells was assessed by Rao et al. (2018) [76]. Click Chemistry reaction was achieved from the previously obtained *O*-propargylated flavones and alkyl or aryl azides. The in vitro screening against MCF-7 cancer cells identified hybrids **35g**, **35i** and **35j** as the most promising, with IC_50_ values of 17.9, 14.2 and 19.1 μM, respectively. When comparing the IC_50_ values of the most promising compounds with other analogues, we suggest that the presence of EWG on C-4 of the benzene ring allied to an unsubstituted R_1_ of the flavone scaffold is associated with an increase in the cytotoxic activity (Figure 13).

Based on the ability of wogonin, a natural flavone, to selectively inhibit cyclin-dependent kinase 9 (CDK9), Bian et al. (2018) synthesized a series of proteolysis-targeting chimeras (PROTACs) targeting CDK9 by recruiting ubiquitin E3 ligase cereblon (CRBN) [77]. Wogonin-based PROTACs **36a**–**36d** (Figure 13) were obtained from conjugation of a *O*-propargylated wogonin with azido-pomalidomide derivatives with a different length of alkyl chain by a triazole linker obtained following the Click Chemistry approach in the presence of copper iodide, hexamethylphosphoramide (HMPA) and *N*,*N*-diisopropylethylenediamine (DIPEA), with excellent yields (92–95%). To perform SAR studies, structure-related hybrids with alkyl chains as the linker instead of triazole were also obtained. Western blotting assays showed that hybrids **36a**–**36d** could selectively downregulate the intracellular CDK9 level. Interestingly, compounds with triazole linker **36a**–**36d** were more potent than structure-related compounds with alkyl chains, suggesting the importance of the triazole ring for this activity. Compounds **36b** (IC_50_ = 15 µM) and **36c** (IC_50_ = 17 µM) were found to inhibit the CDK-9 rich cell line MCF-7 at a lower concentration than wogonin (IC_50_ = 30 µM). Moreover, despite having lower activity than wogonin (IC_50_ = 198 nM), hybrid **36c** showed a selective degradation of CDK9 in a nanomolar concentration (IC_50_ = 523 nM). Further studies revealed that **36c** induced apoptosis in MCF-7 cells in a dose-dependent manner and has low activity in the CDK-9 low-expressed cell line L02 (IC_50_ > 100 µM). Additionally, **36c** decreased the levels of Mcl-1, a prosurvival protein [77]. When comparing biological data of compounds **36a**–**36d**, we suggest that the length of the alkyl chain linker between the triazole and pomalidomide moieties influences the CDK9 degradation effect, the presence of a seven carbon atom alkyl chain linker being the most favorable for the activity.

Flavone derivatives **37a**–**37g** (Figure 13) of apigenin-7-methyl ether were synthesized by CuAAC reaction between *O*-propargylated flavone and substituted azides. These compounds exhibited antiproliferative activity against ovarian cancer cell lines SKOV3, OVCAR-3 and Caov-3 (10 < IC_50_ < 40 µM) [78]. Hybrid **37a** showed the highest antiproliferative activity against all cancer cells (IC_50_ = 10, 15 and 20 µM for SKOV3, OVCAR-3 and Caov-3, respectively) and was found to induce apoptosis on SKOV3 cells through ROS-mediated alterations in mitochondrial membrane potential (MMP), and modulated the expression of Bcl-2 and Bax proteins [78].

Considering the antitumor potential of chrysin, and aiming to explore the influence of chrysin hybridization, Noole et al. (2021) synthesized a series of chrysin hybrids linked through the 1,2,3-triazole ring, which were further tested against four cancer cell lines, namely, PC3, prostate PC3-PSMA, MCF-7 and bladder UM-UC-3 [79]. Firstly, OH-7 of chrysin was protected with benzyl or ethyl groups. After the propargylation of OH-5, the Click Chemistry reaction with different azides was achieved. Eleven compounds (**38a**–**38k**, Figure 13) were shown to have good-to-moderate activity against at least one cancer cell (10.8 < IC_50_ < 93.7 µM). Interestingly, **38e**, **38f**, **38i** and **38k** showed selective activity against PC-3 (IC_50_ = 51.4, 84.7, 19.2 and 57.9 µM, respectively), whereas **38c** was selective against UM-UC-3 (IC_50_ = 72.7 µM). Compound **38b**, with a benzyl group on C7 and a non-substituted phenyl ring linked to the 1,2,3-triazole, was found to exert the highest activity against all cancer cells tested (10.8 < IC_50_ < 43.8 µM), being more active than DOX for PC-3 and MCF-7. Flow cytometry analysis showed that **38b** promotes G2/M cell cycle arrest in MCF-7 cells [79].

### 2.5. 1,2,3-Triazole-Linked Flavonol Hybrids

**Figure 14:**
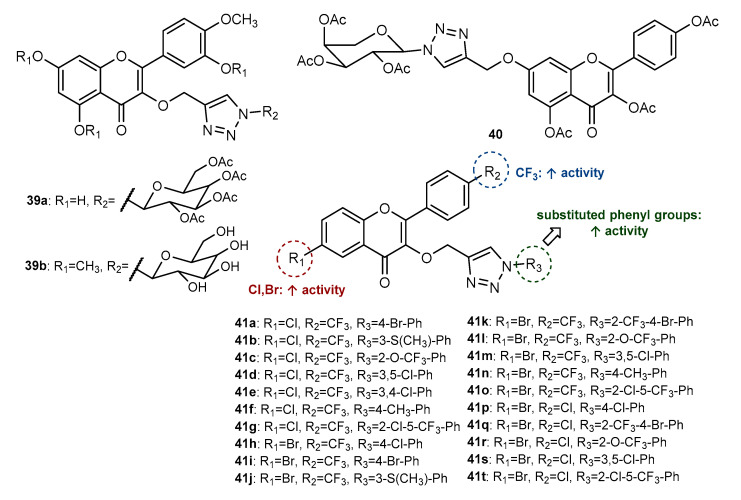
Flavonol hybrids **39**–**41** with antitumor activity.

In addition to the glycosyl flavone **34** previously described in Section 2.4, Wang et al. (2018) identified two glycosyl flavonols with antiproliferative activity (**39a**–**39b**, Figure 14) [75]. Hybrid **39a** displayed moderate activity against all tested cancer cells (IC_50_ = 36.67, 30.56 and 43.42 µM for Hela, HCC1954 and SK-OV-3, respectively), while **39b** had moderate activity for Hela and HCC1954 cells (IC_50_ = 53.33 and 39.79 µM). Specifically, **39a** was found to be more effective for HCC1954 than the positive control *cis*-platin (IC_50_ = 33.57 µM).

Considering that telomerase is widely expressed in human tumors and tumor-derived cell lines, whereas in the normal stem cell this enzyme activity is proportionally low, the inhibition of this enzyme is a strategy for anticancer drug discovery [80]. Fan et al. (2019) reported the synthesis and telomerase inhibitory potential of 1,2,3-linked flavonol-glycosyl hybrid **40** (Figure 14) [81]. The synthesis was performed by Click Chemistry, with previously obtained *O*-propargylated flavonol and glycosyl azide. Compound **40** showed effective telomerase inhibitory activity against HeLa cells (IC_50_ < 50 μM), higher than reference baicalin (IC_50_ > 100 μM). Moreover, hybrid **40** displayed moderate cytotoxic activity against A549, HepG2, HeLa, MGC-803 and gastric SGC-7901 cancer cells (83.183 < IC_50_ < 95.842 μM), higher than the positive control 5-FU (95.172 < IC_50_ < 110.164 μM). Interestingly, **40** revealed no cytotoxic effect against two normal cells (Hacat and human bronchial epithelial BEAS-2B), with IC_50_ values > 200 μM, showing selectivity for cancer cells. Molecular docking studies and molecular dynamics analyses allowed us to conclude that **40** has similar interactions with telomerase to those observed for BIBR1532, a highly specific inhibitor of this enzyme [81].

Znati et al. (2021) reported the synthesis of a series of flavonol-linked 1,2,3-triazole conjugates and their cytotoxic activity against HCT-116, MCF-7 and OVCAR-3 (human ovarian carcinoma) cancer cells [82]. Intermediate flavonols were obtained from acetophenone and benzaldehyde building blocks by Claisen–Schmidt condensation followed by Algare–Flynne–Oyamada in one step. Interestingly, after the *O*-propargylation of the flavonols, authors performed the CuAAC reaction in different conditions, namely, at room temperature, conventional heating, and MW irradiation, which provided the final compounds with higher yields and a lower reaction time (from 48 h in room temperature to 5 min using MW). A series of 20 compounds (**41a**–**41t**, Figure 14) were found to exert some activity in at least one cancer cell, **41d**, **41e** and **41o** being the most active against HCT-116 and **41b** and **41n** the most active against OVCAR-3, with IC_50_ lower than 3.0 µM. These compounds were further submitted to molecular docking studies within the active site of topoisomerase IIα. It was found that the presence of CF_3_ group on R_2_ allied to halogen groups on R_1_ greatly contributes for cytotoxic activity, as well as the presence of substituted phenyl groups linked to the triazole ring, comparing to unsubstituted phenyl group [82].

### 2.6. 1,2,3-Triazole-Linked Isoflavone Hybrids

**Figure 15:**
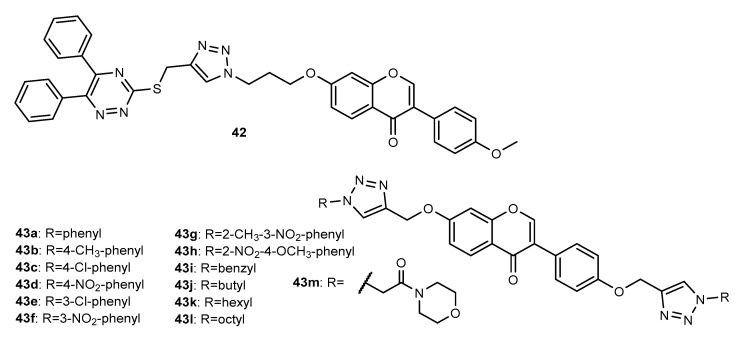
Isoflavone hybrids **42**–**43** with antitumor activity.

Besides the 5,6-diaryl-1,2,4-triazine-chalcone derivative **4**, Fu et al. (2017) also reported the synthesis and antiproliferative activity of a 5,6-diaryl-1,2,4-triazine-isoflavone hybrid **42** (Figure 15) [53]. This compound displayed potent activity against PC-3 cancer cell line (IC_50_ = 10.23 µM), higher than 5-FU (IC_50_ = 12.87 µM), without significant activity against MGC-803 and EC-109 (IC_50_ > 100 µM), showing selectivity for PC-3, among other cancer cells.

Based on the antitumor potential of natural isoflavone daidzein, and trying to overcome its pharmacokinetic limitations, such as low oral bioavailability and water insolubility through the incorporation of a heterocyclic ring, Yerrabelly et al. (2020) synthesized a series of daidzein bridged bis-[1,2,3]-triazole derivatives **43a**–**43m** (Figure 15), based on the Click Chemistry approach from bis-*O*-propargylated daidzein and substituted azides [83]. All the obtained compounds showed to be active against A549, HeLa and MDA-MB-231 cancer cell lines (0.14 < GI_50_ < 17.8 µM), while daidzein showed GI_50_ values from 1.48 to 16.7 µM. Compounds **43i** (GI_50_ = 0.23 µM), **43j** (GI_50_ = 0.24 µM) and **43l** (GI_50_ = 0.14 µM) were identified as the most potent compounds for A549, HeLa and MDA-MB-231 cells, respectively.

## 3. 1,2,3-Triazole-Linked Flavonoid Hybrids with Antimicrobial Activity

**Figure 16:**
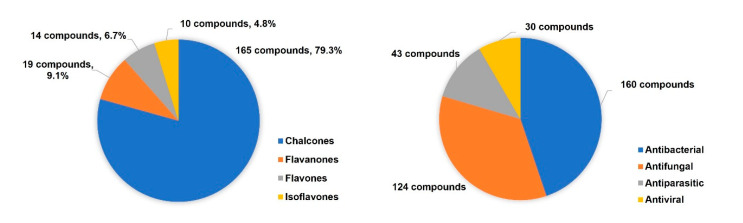
Distribution of the flavonoid hybrids according to the reported antimicrobial activity.

More than 200 flavonoid hybrids linked by the 1,2,3-triazole ring with antimicrobial activity have been reported since 2017, the majority being chalcone derivatives (Figure 16). Most of the described flavonoid hybrids showed antibacterial and antifungal activity. Nevertheless, some reports about flavonoids with antiparasitic and antiviral activity can also be found in literature (Figure 16). It should be noted that several compounds simultaneously exhibited antibacterial, antifungal, and antiparasitic activities.

### 3.1. 1,2,3-Triazole-Linked Chalcone Hhybrids

**Figure 17:**
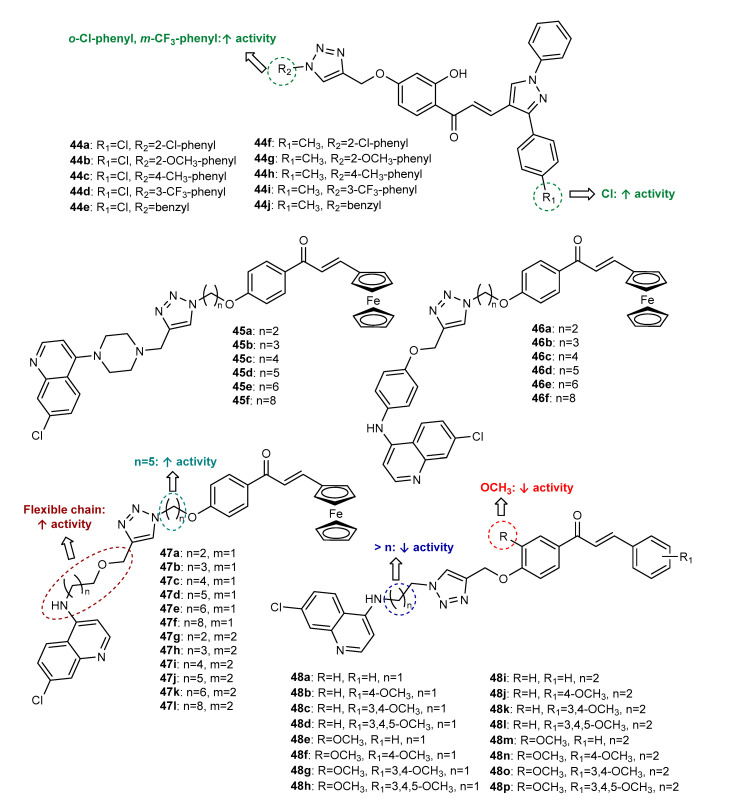
Chalcone hybrids **44**–**48** with antimicrobial activities. (**44a**–**44j**: antibacterial and antifungal activities; **45a**–**45f**, **46a**–**46f**, **47a**–**47l**: antitubercular and antiparasitic activities; **48a**–**48p**: antiparasitic activity.)

Ashok et al. (2017) reported the synthesis and antimicrobial activity for a series of ten pyrazole-chalcones conjugated with a 1,2,3-triazole ring (**44a**–**44j**, Figure 17), since both of these scaffolds are referenced for antimicrobial activity [84]. The CuAAC reaction occurred from *O*-propargylated pyrazole-chalcones and aryl azides. The tested compounds displayed moderate antibacterial activity against *B. subtilis* (MIC = 3.125–50 µg/mL), *S. aureus* (MIC = 3.125–50 µg/mL), *E. coli* (MIC = 3.125–50 µg/mL) and *P. vulgaris* (MIC = 6.25–50 µg/mL), as well as antifungal activity against *A. niger* (MIC = 6.25–50 µg/mL) and *C. albicans* (MIC = 6.25–50 µg/mL). Among them, **44a**, with chlorophenyl groups linked to both the pyrazole and triazole nucleus, displayed the highest antibacterial efficacy (*B. subtilis*, *S. aureus* and *E. coli:* MIC = 3.125 µg/mL; *P. vulgaris:* MIC = 6.25 µg/mL; reference drug gentamicin: MIC = 1.56–3.125 µg/mL). Compound **44a** also showed the highest antifungal activity against both fungal strains with an MIC value of 6.25 µg/mL (reference drug fluconazole: MIC = 1.56–3.125 µg/mL). Hybrid **44d**, bearing a *p*-chlorophenyl group on the pyrazole ring and a *m*-(trifluoromethyl)phenyl group on the benzene ring attached to the 1,2,3-triazole (Figure 17), also displayed potent antibacterial and antifungal activities (MIC = 6.25–12.5 µg/mL), suggesting the role of EWG in these moieties for the antimicrobial activity.

Considering the antiplasmodial potential of aminoquinoline derivatives, namely, the conjugate ferroquine (7-chloroquinoline-ferrocene), which was in clinical trials for the treatment of malaria, and also the antiplasmodial potential of chalcones, three different series of 4-aminoquinoline-ferrocenyl-chalcone conjugates, **45a**–**45f**, **46a**–**46f** and **47a**–**47l** (Figure 17), were synthesized by Click Chemistry reaction between *O*-alkyl azide ferrocenyl-chalcones and substituted propargylated quinoline derivatives [85]. All compounds showed good antiplasmodial activity against the chloroquine (CQ)-resistant W2 strain of *P. falciparum* with IC_50_ values ranging from 0.37 to 5.08 µM. Compound **47j** was found to be the most potent, showing an IC_50_ of 0.37 µM. Interestingly, no cytotoxicity was observed for these compounds, suggesting their potential selective cytotoxic effect for *P. falciparum*. Compounds with a flexible chain as a substituent on the quinoline core showed an improved antiplasmodial activity (**47a**–**47l**, 0.37 < IC_50_ < 2.92 µM), compared to the compounds with piperazine (**45a**–**45f**, 2.55 < IC_50_ < 5.08 µM) or aminophenol (**46a-46f**, 1.16 < IC_50_ < 4.98 µM) functionalities. All compounds were also screened for antitubercular activity against the mc^2^6230 strain of *Mycobacterium tuberculosis* [86]. Most of the tested compounds displayed moderate-to-weak antitubercular activity (37 < MIC_99_ < 100 µM). The conjugates with acyclic flexible chains as substituents on the quinoline ring (**47a**–**47l**) exhibited better activity profiles compared with the ones with cyclic substituents such as piperazine (**45a**–**45f**) or aminophenol (**46a**–**46f**). Among them, **47j** showed the highest activity, with MIC_99_ = 30 µM (standard drug isoniazid: MIC_99_ = 0.15 µM).

In addition to 4-aminoquinoline-chalcone conjugates, **45a**–**45f**, **46a**–**46f** and **47a**–**47l**, other structure-related compounds (**48a**–**48p**, Figure 17) with antimalarial activity against the CQ-resistant W2 strain of *P. falciparum* were identified by Kumar et al. (2017) [87]. The CuAAC reaction between *O*-propargylated chalcones and 4-aminoquinoline *N*-alkyl azide derivatives gave rise to **48a**–**48p** with good-to-excellent yield (79–93%). Among the tested compounds, **48b** and **48c** showed the most promising activity with IC_50_ = 114.4 and 140.9 nM, respectively, being more potent than the reference drug CQ (IC_50_ = 150 nM). Interestingly, these compounds were further screened for cytotoxic activity against HeLa cells, showing selective activity for *P. falciparum*. SAR studies revealed that the presence of a methoxy group on A ring of the chalcone scaffold decreases antiplasmodial activity. Moreover, the increase in the size of the alkyl chain between the triazole ring and quinoline resulted in significant loss of the activity (Figure 17).

**Figure 18:**
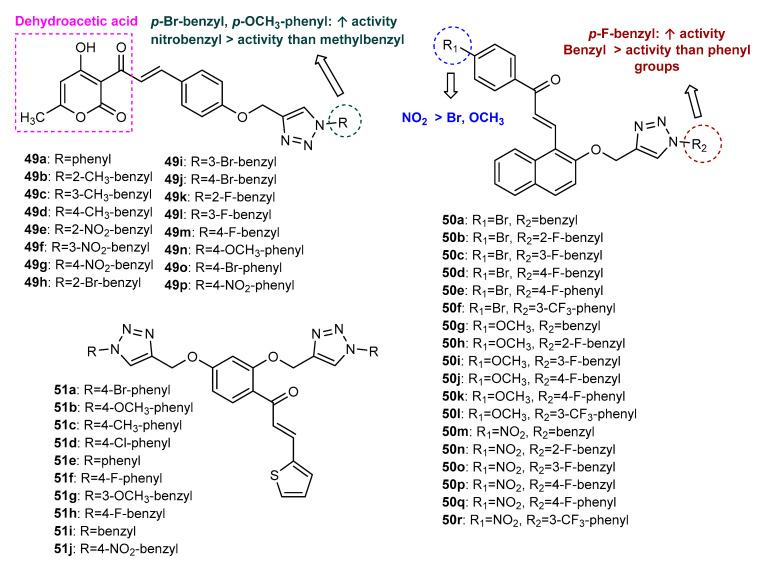
Chalcone hybrids **49a**–**49p**, **50a**–**50r** and **51a**–**51j** with antibacterial and antifungal activities.

Using dehydroacetic acid (DHA), a heterocyclic compound with fungicide and bactericide activities [88], as inspiration, a propargylated DHA-based chalcone was synthesized and used as intermediate for the synthesis of a series of 1,2,3-triazole-linked DHA-chalcone hybrids (**49a**–**49p**, Figure 18) by Lal et al. (2018) [89]. The Click Chemistry reaction between *O*-propargylated chalcone and aryl azides was performed in the presence of cellulose-supported copper iodide nanoparticles in water, under heating, to yield compounds **49a**–**49p** in high yields (75–87%). All compounds were screened for antimicrobial activity against four bacterial strains (*Staphylococcus epidermidis*, *Bacillus subtilis*, *Escherichia coli* and *Pseudomonas aeruginosa*) and two fungal strains (*Aspergillus niger* and *Candida albicans*) [89]. Most of the synthesized compounds showed an improvement of antimicrobial activity compared to DHA (MIC = 0.0372 µM/mL) and DHA-based chalcone used as intermediate (MIC = 0.0202–0.0404 µM/mL). Among them, **49j** displayed the highest activity against *E. coli* (MIC = 0.0034 µM/mL), *B. subtilis* (MIC = 0.0034 µM/mL) and *A. niger* (MIC = 0.0060 µM/mL), better than the reference drugs ciprofloxacin (MIC = 0.0047 µM/mL) and fluconazole (MIC = 0.0102 µM/mL). Compound **49n** also displayed excellent activity against all the tested bacterial strains, being the most potent for *S. epidermidis* and *P. aeruginosa* with an MIC value of 0.0034 µM/mL (ciprofloxacin: MIC = 0.0047 µM/mL). This hybrid also showed the highest activity against *C. albicans* (MIC = 0.0034 µM/mL), better than fluconazole (MIC = 0.0051 µM/mL). After analysis of the antimicrobial data, several SAR conclusions were made as illustrated in Figure 18. In general, the presence of substituents on the benzene ring linked to the triazole moiety mostly provides higher antibacterial and antifungal activities, the compounds containing *p*-bromobenzyl or *p*-methoxyphenyl groups being the most potent. Moreover, the presence of nitrobenzyl groups attached to the triazole moiety is associated with a higher antibacterial and antifungal activity than those observed for substituents with methylbenzyl groups. Docking studies of hybrid **49j** into the active site of *E. coli* DNA gyrase topoisomerase II, allowed us to predict that all three parts of the molecule, i.e., DHA, phenoxy ring and triazole, are important for antibacterial activity [89].

The same research group also reported the antimicrobial activity of a series of 18 chalcone hybrids (**50a**–**50r**, Figure 18), obtained by the conjugation of naphthaldehyde-chalcone alkynes with aryl azides by CuAAC, following the same procedure as described above [90]. The antimicrobial screening revealed that most of the triazole hybrids exhibited significant efficacy against the bacterial (MIC = 0.0032–0.0522 µmol/mL) and fungal (MIC = 0.0032–0.0526 µmol/mL) strains tested, being more potent than the synthetic naphthaldehyde-chalcone alkynes used as synthetic intermediates [90]. Compounds **50j** and **50p** showed the highest activity against *E. coli*, with MIC values of 0.0032 µmol/mL, better than ciprofloxacin (MIC = 0.0047 µmol/mL). Hybrid **50d** displayed the highest activity against *B. subtilis* (MIC = 0.0058 µmol/mL), whereas for *P. aeruginosa*, **50o** and **50p** were found to be the most active (MIC = 0.0063 µmol/mL). Compound **50p** also displayed the highest activity against bacterial strain *S. epidermidis* (MIC = 0.0032 µmol/mL), better than ciprofloxacin (MIC = 0.0047 µmol/mL), and fungal strains *C. albicans* and *A. niger* (MIC = 0.0032 µmol/mL), better than fluconazole. Docking simulation showed that compound **50p** binds effectively into the active site of *E. coli* topoisomerase II DNA gyrase B. By comparing the antibacterial and antifungal activities of the different compounds, it seems that the compounds with a nitro group on the chalcone scaffold showed higher activity than compounds bearing bromine or methoxy groups. Moreover, compounds with a benzyl group attached to the triazole ring showed higher activity than compounds with a phenyl group, the analogues with the *p*-fluorobenzyl group being the most potent from the series (Figure 18). 

Kiran et al. synthesized a series of 10 thiophene-chalcone hybrids bearing a *bis*-triazole ring with phenyl (**51a**–**51f)** or benzyl (**51g**–**51j)** substituents (Figure 18) [91]. CuAAC reaction was performed between previously obtained *O*-propargylated thiophene-chalcone and aryl azides, with 76–90% yield. All the synthesized analogues exhibited antibacterial effects against Gram-positive (*S. aureus* and *B. cereus*, zones of inhibition between 1.2 and 6.0 mm at 200 µg/mL) and Gram-negative (*E. coli* and *P. aeruginosa*, zones of inhibition between 2.0 and 6.0 mm at 200 µg/mL) bacteria and *C. albicans* (zones of inhibition between 2.0 and 4.0 mm at 200 µg/mL), this effect being lower than the standard drug streptomycin (zones of inhibition between 5.5 and 10.0 mm at 200 µg/mL).

**Figure 19:**
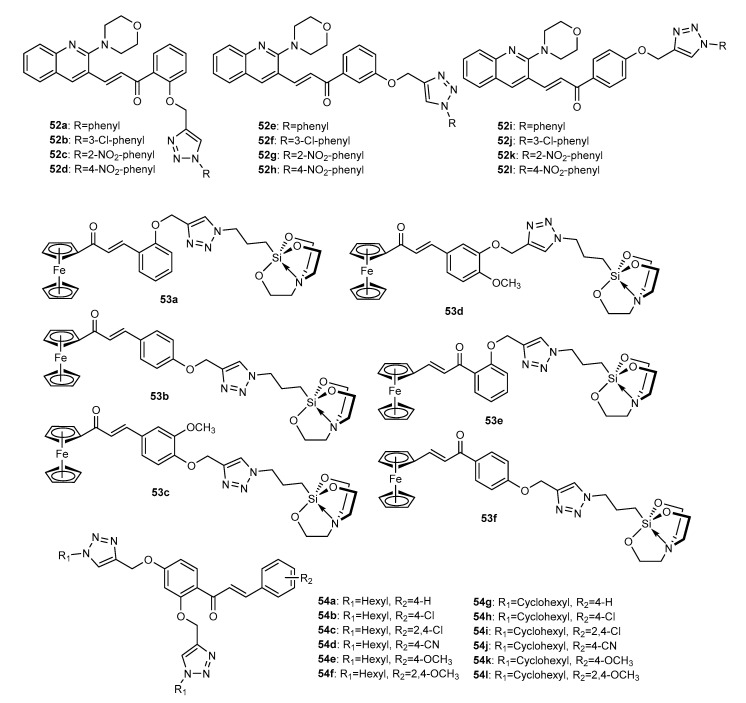
Chalcone hybrids **52**–**54** with antimicrobial activities. (**52a**–**52l**, **53a**–**53f** and **54a**–**54l**: antibacterial and antifungal activities; **53a**–**53f**: antiparasitic activity.)

A series of 12 morpholino-quinolinyl chalcones conjugated with 1,2,3-triazole ring **52a**–**52l** (Figure 19) were synthesized by Pujari et al. (2018) and further screened for their antimicrobial activity against a panel of bacterial and fungal strains [92]. After the synthesis of intermediate *O*-propargylated chalcone, the CuAAC reaction with aryl azides occurred in the presence of copper sulphate and sodium ascorbate in DMF/water, under MW irradiation, giving the final compounds in excellent yields. Interestingly, authors tested the influence of several solvents for the reaction and realized that the mixture of DMF/water give the highest yields and the lowest time of reaction. Compounds showed moderate activity against *B. faecalis*, *S. aureus*, *K. pneumonia* and *E. coli* bacterial strains, with zones of inhibition of 9.8–32.0 mm at 100 μg/mL (positive control ampicillin: 20.2–35.8 mm). Hybrids **52a**–**52l** also showed antifungal activity against *Aspergillus niger* and *Candida metapsilosis*, showing an inhibition zone of 8.7–21.3 mm at 500 μg/mL (positive control griseofulvin: 13.4–18.6 mm).

Considering the biological potential of ferrocenyl chalcones and the silatranyl nucleus and aiming to combine these pharmacophores in a single molecule, Singh et al. (2019) reported the synthesis and antimicrobial activity of a series of six ferrocene chalcone-organosilatrane conjugates (**53a**–**53f**, Figure 19) linked by a 1,2,3-triazole ring on the A or B ring of the chalcone scaffold [93]. The CuAAC reaction between previously obtained ferrocenylchalcones and corresponding azides occurred in the presence of triethylamine and bromotris (triphenylphosphine) copper (I) in THF, under reflux, with 82–94% yield. Except for **53e**, all tested compounds showed moderate-to-low antibacterial (125 < IC_50_ < 250 µM) and antifungal (31.25 < IC_50_ < 250 µM) activity against *E. coli*, *S. aureus*, *E. faecalis*, *V. cholera*, *H. influenza* and *L. monocytogenes* bacterial strains and *C. krusei*, *C. albicans*, *C. tropicalis*, *C. parapsilosis*, *C. kyfer*, *C. neoformans* and *C. glabrata* fungi strains. Compounds also showed moderate-to-potent antiparasitic activity against *T. vaginalis* and *G. lamblia* after 24 or 48 h incubation (IC_50_ = 0.57–130 µM). Among them, **53e** displayed excellent activity against *G. lamblia* after 24 h incubation (IC_50_ = 0.57 µM), whereas **53a** displayed the highest activity after 48 h incubation (IC_50_ = 1.57 µM). For *T. vaginalis*, **53b** showed the highest activity (IC_50_ = 57.72 and 22.90 µM after 24 h and 48 h incubation, respectively). It is worth mentioning that all bioactive compounds were predicted to have adequate ADMET properties.

Sunitha et al. (2020) synthesized 12 chalcone derivatives (**54a**–**54l**, Figure 19) and screened their antimicrobial activity [94]. The CuAAC reaction was performed in the *O*-propargylated acetophenone with excellent yields. The Claisen–Schmidt condensation of previously obtained triazole acetophenones and different substituted benzaldehydes gave rise to the final compounds **54a**–**54l**. All compounds displayed moderate antibacterial activity against both Gram-positive (*M. luteus*, MRSA, *B. subtilis*, *B. cereus)* and Gram-negative bacteria (*P. aeruginosa*, *K. pneumonia*, *E. coli* and *P. vulgaris*), showing a zone of inhibition of 14–37 mm at 100 µg/mL (positive control gentamicin: 30–34 mm at 100 µg/mL). Except for **54d** and **54j**, all compounds showed antifungal activity against *Microsporum canis*, *Microsporum gypseum* and *Epidermophyton floccosum* at 100 µg/mL (zone of inhibition: 6–28 mm) comparing to the positive control nystatin (zone of inhibition: 23–28 mm at 100 µg/mL).

**Figure 20:**
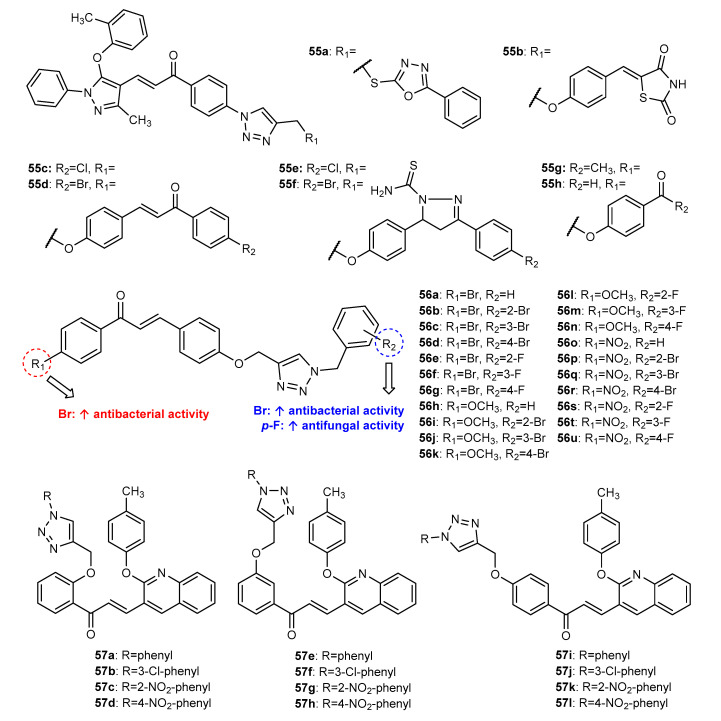
Chalcone hybrids **55**–**57** with antimicrobial activities. (**55a**–**55h**: antibacterial activity; **56a**–**56u** and **57a**–**57l**: antibacterial and antifungal activities.)

Nagaraja et al. (2020) reported the synthesis and antimicrobial activity of a series of eight pyrazole chalcones bearing a 1,2,3-triazole ring **55a**–**55h** (Figure 20), including two chalcone dimers **55c** and **55d** [95]. The Click Chemistry reaction between azide pyrazole-chalcone and propargylated substituents achieved the final compounds with 61–77% yield. All compounds were shown to inhibit Gram-positive bacteria *S. aureus* (NCIM-5021) and *B. subtilis* (NCIM-2197) and Gram-negative bacteria *E. coli* (NCIM-2931) and *P. aeruginosa* (NCIM-2036) at 100 μg/mL, with zones of inhibition ranging between 10 and 20 mm, compared to the positive control ciprofloxacin (zone of inhibition ranging from 22 to 24 mm at the same concentration).

The antimicrobial potential of 21 chalcone derivatives bearing a 1,2,3-triazole (**56a**–**56u**, Figure 20) against a panel of bacterial and fungal strains was assessed by Yadav et al. (2021) [96]. All compounds were found to significantly inhibit four bacterial strains, namely, *S. epidermidis* MTCC 6880, *B. subtilis* MTCC 441, *E. coli* MTCC 16521 and *P. aeruginosa* MTCC 424 (MIC = 0.0113–0.0495 μmol/mL). When comparing the results, it can be inferred that the presence of a bromobenzene A ring in the chalcone scaffold as well as the bromophenyl group linked to the 1,2,3-triazole moiety is beneficial for antibacterial activity. Moreover, chalcone hybrids showed higher activity than intermediate chalcone azides, confirming the advantage of hybridization. Concerning antifungal activity, all compounds were active against two resistant fungal strains (*A. niger* MTCC 8189 and *C. albicans* MTCC 227), with MIC values ranging between 0.0063 and 0.0406 μmol/mL. It should be noted that compounds **56g** and **56u** showed a higher activity for *C. albicans* (MIC = 0.0063 and 0.0068 μmol/mL) than reference drug fluconazole (MIC = 0.0102 μmol/mL), and the presence of *p*-fluorophenyl group is generally associated with better activity. Further, compounds **56q** and **56g** were docked into the active pocket of *E. coli* topoisomerase II DNA gyrase B and *C. albicans* lanosterol 14α-demethylase, respectively, confirming the high efficacy of the compounds.

The synthesis of a series of quinoline-based chalcone hybrids, **57a**–**57l** (Figure 20), was reported by Vishnuvardhan et al. (2021) [97]. The influence of different catalysts and solvents, as well as the use of conventional methods and MW heating for the CuAAC reaction was also presented, with maximum yield and lower time for the reaction carried under MW irradiation in the presence of CuI in DMF/water. Among them, compounds **57d**, **57h** and **57i** displayed the highest antibacterial activity against *B. faecalis*, *S. aureus*, *K. pneumonia* and *E. coli*, and **57d**, **57h** and **57k** presented the highest activity against *A. niger* and *Candida metapsilosis* fungal strains. The remaining compounds were found to display low-to-moderate activity.

### 3.2. 1,2,3-Triazole-Linked Flavanone Hybrids

**Figure 21:**
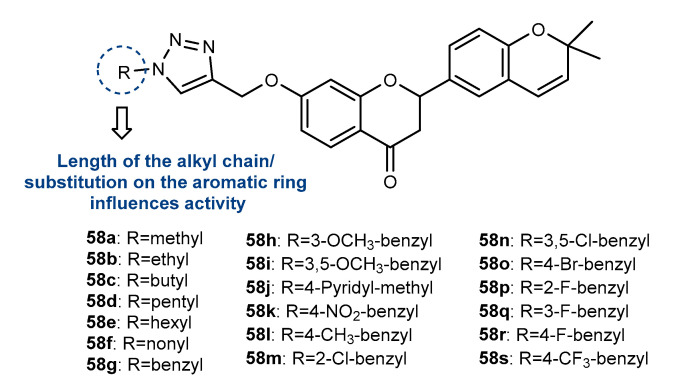
Flavanone hybrids **58a**–**58s** with antiviral activity.

Zhang et al. (2021) reported the synthesis of a series of flavanone derivatives (**58a**–**58s**, Figure 21) and their antiviral activity against hepatitis C virus (HCV) [98]. After the synthesis of intermediate *O*-propargylated flavanone, the Click Chemistry step was performed with different azides, giving compounds **58a**–**58s** with 56–80% yield. Among them, compounds **58d**, **58e**, **58m**, **58o** and **58r** showed significant HCV inhibitory activity. When comparing the results, it was verified that the length of the alkyl chain may affect the activity, as well as the substitution of the aromatic ring linked to 1,2,3-triazole. Compounds **58m** and **58r** were found as the most active, with IC_50_ values of 5.285 and 9.004 µM, respectively. Moreover, these compounds inhibited the HCV production in a dose-dependent manner, being the activity comparable with the positive control at 30 µM. Complementary studies showed that **58m** and **58r** inhibited the entry of HCV in the cell surface by 34% and 52% at 10 µM, respectively [98].

### 3.3. 1,2,3-Triazole-Linked Flavone Hybrids

**Figure 22:**
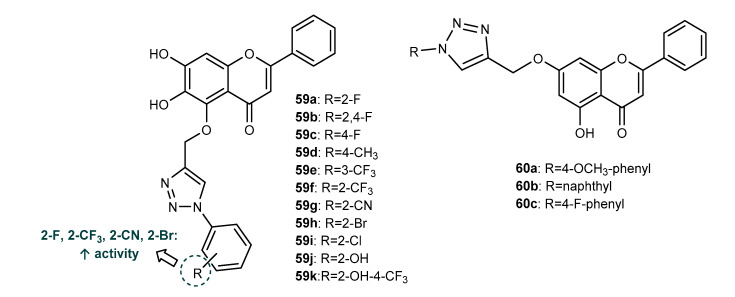
Flavone hybrids **59**–**60** with antimicrobial activities. (**59a**–**59k**: antiviral activity; **60a**–**60c**: antibacterial activity.)

Based on baicalein, a natural flavone known for its anti-inflammatory and antiviral activities, Zhang et al. (2019) synthesized a series of baicalein hybrids, **59a**–**59k** (Figure 22), which were evaluated for their ability to prevent infection by the respiratory syncytial virus (RSV) [99]. The step of Click Chemistry between *O*-propargylated baicalein and aryl azides was catalyzed by copper sulphate and sodium ascorbate in a mixture of *t*-butanol/water at 45 °C under sonication, affording compounds **59a**–**59k** with 80–91% yield. Firstly, all the synthesized compounds were screened for their ability to prevent in vitro RSV replication on bronchial epithelial BEAS-2B cells infected with RSV. Although most of the compounds displayed moderate activity (MIC = 50–100 µM), compounds **59a**, **59f**, **59g** and **59h**, with MIC values of 3.0, 2.5, 3.25 and 3.12 µM, showed the highest activity, comparable to the standard drug ribavirin (MIC = 2.7 µM). SAR analysis showed that the presence of substituents on *ortho*-position in the benzene ring linked to the triazole moiety is desirable for the activity (Figure 22). Further studies using RSV-infected BEAS-2B cells and an in vivo mice model showed that the most potent compound, **59f**, inhibits replication of RSV through the activation of the interferon (IFN) signaling pathway and prevented inflammation of pulmonary airways through down-regulation of ROS generation and pro-inflammatory cytokines expression [99].

In order to obtain chrysin derivatives with promising antimicrobial activity and to improve their bioavailability, Bhowmik et al. (2021) synthesized chrysin derivatives linked through the 1,2,3-triazole ring, **60a**–**60c** (Figure 22), by CuAAC from *O*-propargylated chrysin and aryl azides [100]. All derivatives displayed low antibacterial activity against the planktonic form of *E. coli* MTCC 40 (30.68% inhibition at 100 µg/mL for **60a**, 11.36% inhibition at 450 µg/mL for **60b** and 15.27% inhibition at 120 µg/mL for **60c**), lower than the positive controls chrysin (18.29% inhibition at 70 µg/mL) and gentamycin (90.76% inhibition at 0.5 µg/mL). These compounds were also screened against the biofilm form of *E. coli*, with an inhibition of 17.86% at 100 µg/mL for **60a**, 31.43% inhibition at 450 µg/mL for **60b** and 93.57% inhibition at 120 µg/mL for **60c**, whereas the positive controls chrysin and gentamycin showed 33.57% inhibition at 70 µg/mL and 99.07% inhibition at 0.5 µg/mL, respectively. As compound **60c** showed potential antibiofilm activity, further studies using the fluorescence measurement technique were performed, a retarded formation of biofilm being observed in comparison with chrysin [100]. Moreover, the motility of *E. coli* was significantly restricted by **60c** at sub-inhibitory concentrations, it being higher than chrysin. Compound **60c** also displayed higher bioavailability than chrysin and good cell permeability and drug-likeness in in silico ADME analysis [100].

### 3.4. 1,2,3-Triazole-Linked Isoflavone Hybrids

**Figure 23:**
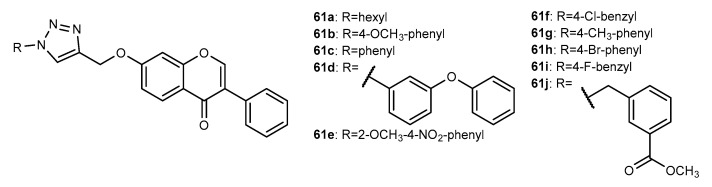
Isoflavone hybrids **61a**–**61j** with antibacterial and antifungal activities.

Ten isoflavone conjugates, **61a**–**61j** (Figure 23), with antimicrobial activities were reported by Yerrabelly et al. (2020) [101]. Excepting for **61i**, all compounds showed some activity against *B. subtilis*, with zones of inhibition between 0.1 and 0.4 cm at 500 µg/mL, whereas the positive control streptomycin presented an inhibition zone of 1.9 cm at the same concentration. All compounds were shown to be ineffective against *E. coli*. Regarding antifungal activity, all compounds were found to inhibit *Sclerotium rolfsii* (inhibition zones between 0.3 and 1.9 cm at 500 µg/mL). Among them, compounds **61a, 61b** and **61d** (1.1–1.9 cm at 500 µg/mL) displayed higher activity than the positive control bavistin (inhibition zone of 0.8 cm at 500 µg/mL). None of the tested compounds were active against *Macrophamina phaseolina*.

## 4. Other Activities

**Figure 24:**
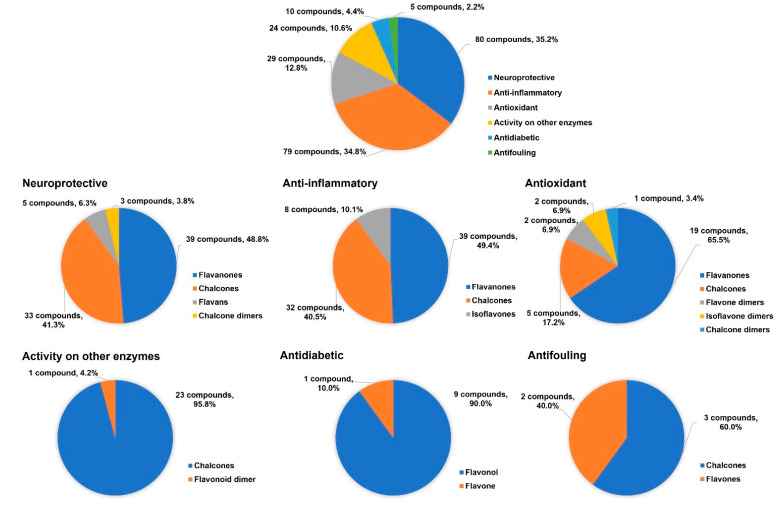
Other activities attributed to 1,2,3-triazole-linked flavonoid hybrids.

Besides antitumor and antimicrobial activities, a total of 163 flavonoid hybrids conjugated by the 1,2,3-triazole ring with other activities, including antidiabetic, neuroprotective, anti-inflammatory, antioxidant, and antifouling, were also reported. Moreover, some of these compounds have been shown to be modulators of other enzymes, namely, carbonic anhydrase (CA), serine proteases and glutatione S-transferase (GST) (Figure 24). The distribution of the different classes of flavonoids is highlighted in Figure 24.

### 4.1. Neuroprotective Activity

Neurodegenerative disorders, which includes Alzheimer’s disease (AD), Parkinson’s disease, amyotrophic lateral sclerosis, Huntington disease, multiple sclerosis, and stroke, are caused by the progressive degeneration of nerve cells in the brain. There are several mechanisms underlying neurodegeneration, namely, oxidative stress, mitochondrial dysfunction, excitotoxicity, neuroinflammation, and protein aggregation, which promotes neuronal apoptosis. Therefore, neuroprotective compounds aim to attenuate or prevent the mechanisms responsible for neurodegeneration [102]. Several natural flavonoids are known for their neuroprotective activities [103,104].

**Figure 25:**
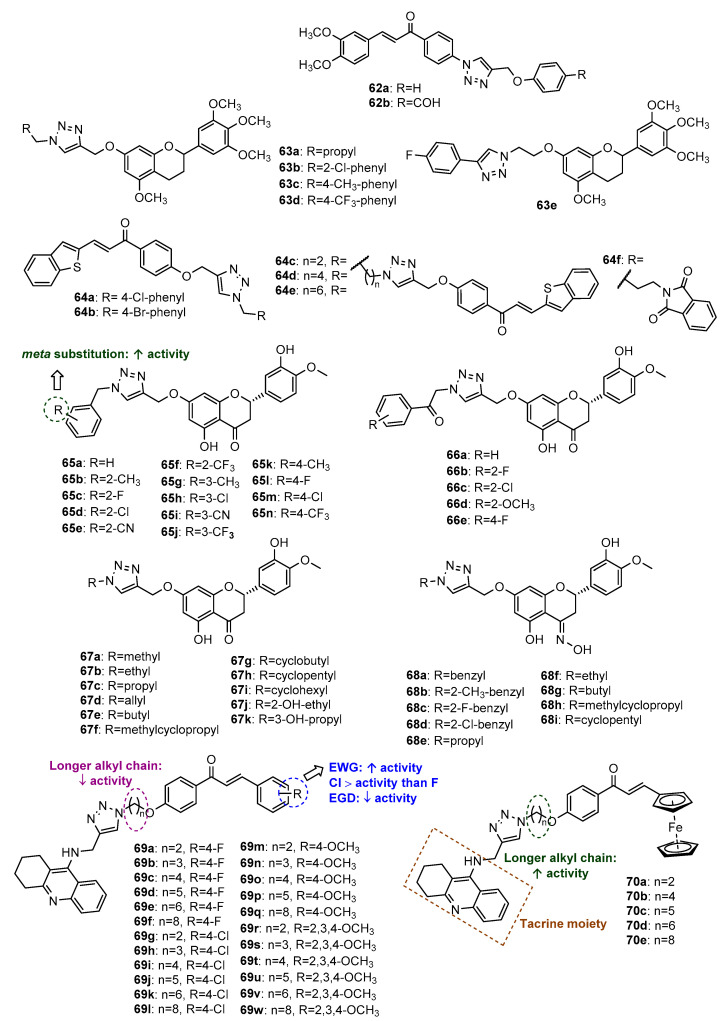
Flavonoid hybrids **62**–**70** with neuroprotective activity.

Chalcone hybrids **62a**–**62b** (Figure 25), reported by Sooknual et al. (2020), were found to display neuroprotective activity through several mechanisms [105]. These compounds were prepared by CuAAC reaction from previously obtained chalcone azide and aryl alkynes. Compounds **62a**–**62b** were found to counteract the reduction in cell viability in neuroblastoma SH-SY5Y cells compared with H_2_O_2_ treatment alone, having similar activity compared to the positive control resveratrol, a well-known antioxidant, with low cytotoxicity toward the normal vero cell line (IC_50_ > 50 μg/mL). These compounds significantly improved the morphology of neurons and increased the cell survival rate of neuronal cells induced by oxidative stress. Moreover, these compounds prevented Aβ oligomer-induced cytotoxicity, similar to positive control resveratrol. Further studies showed that **62a**–**62b** promoted neuroprotection through the SIRT1/2/3-FOXO3a pathway, counteracting H_2_O_2_-induced mitochondrial dysfunction, modulating pro- and anti-apoptotic proteins, as well as upregulating SOD2 mitochondrial antioxidant enzyme.

Among neurodegenerative disorders, AD is one of the most common and the leading cause of dementia in elderly people. This disease is characterized by the accumulation of intracellular neurofibrillary tangles (NFTs) and extracellular deposition of β-amyloid plaques at synapses, which causes the degeneration of neurons and associated loss of synapses and low levels of neurotransmitters in the brain, namely, acetylcholine. Based on the mechanisms underlying AD, there are several possible strategies for the AD treatment, which includes the inhibition of the formation or aggregation of Aβ peptides, thereby preventing the formation of amyloid plaques and the modulation of the acetylcholine levels, through inhibition of acetylcholinesterase (AChE), thus restoring the levels of this neurotransmitter in the synapses [106,107].

Based on antioxidant and neuroprotective activities of the natural flavonoid 7-*O*-galloyltricetiflavan, and considering its instability and low bioavailability, Shi et al. (2020) synthesized a series of flavan hybrids, including 1,2,3-triazole-linked flavan hybrids **63a**–**63e** (Figure 25), which were further evaluated for their ability to prevent AD through several mechanisms [108]. The synthesis of derivatives **63a**–**63d** occurred from *O*-propargylated flavan and aryl azides, whereas derivative **63e** was obtained from flavan azide and the corresponding aryl alkyne. All compounds were found to inhibit Aβ_1-42_ self-induced aggregation (50.94–65.32% at 20 µM), as also described for the parent flavonoid 7-*O*-galloyltricetiflavan (53.11% at 20 µM). Interestingly, **63a**–**63e** displayed a greater acetylcholinesterase (AChE) and butyrylcholinesterase (BuChE) inhibitory effect (0.65 < IC_50_ < 9.46 µM for AChE and 5.77 < IC_50_ < 11.39 µM for BuChE, respectively) than 7-*O*-galloyltricetiflavan (IC_50_ = 76.56 and 47.24 µM for AChE and BuChE, respectively). Among them, **63d** presented the highest activity for both enzymes, being comparable with reference drug donepezil (IC_50_ = 0.134 µM for AChE and IC_50_ = 4.30 µM for BuChE). It seems that the presence of a *para*-fluorinated phenyl group (*p*-CF_3_ for **63d** and *p*-F for **63e**) is beneficial for the anti-AChE and anti-BuChE activities. The most active compound **63d** was further submitted to kinetic, molecular modeling and molecular dynamics studies, which revealed that **63d** could interact with the catalytic active site of BuChE. Moreover, **63d** did not show cytotoxicity against the human SH-SY5Y neuroblastoma cell line, even at the highest concentration tested. Interestingly, this compound was found to increase the SH-SY5Y cell viability for all the tested concentrations, and to protect H_2_O_2_-induced oxidative cell damage in SH-SY5Y cells (78.22%), to a greater extent than trolox (78.22%) at 50 μM, suggesting its neuroprotective effect [108].

Çelik et al. (2021) reported the synthesis of six chalcone derivatives (**64a**–**64f**, Figure 25) and their influence on AChE and BuChE enzymes [109]. All compounds displayed inhibitory activity of both enzymes, with IC_50_ = 7.92–11.72 µM and Ki = 5.88–8.43 µM for AChE and with IC_50_ = 7.79–12.31 µM and Ki = 5.08–11.72 µM for BuChE, **64e** and **64a** being the most active for AChE and BuChE, respectively.

Four series of hesperetin derivatives (**65a**–**65n**, **66a**–**66e**, **67a**–**67k** and **68a**–**68i**, Figure 25) were evaluated for their anti-Alzheimer activity by Wang et al. (2021) [110]. These compounds were also screened for anti-inflammatory activity (see subchapter 4.2) [111]. These compounds were firstly screened for their ability to inhibit cholinesterase enzymes AChE and BuChE, in order to find selective inhibitors of BuChE. Among them, **65h** (IC_50_ = 3.08 µM), **65m** (IC_50_ = 4.61 µM), **66d** (IC_50_ = 5.51 µM) and **66e** (IC_50_ = 6.18 µM) showed higher activity for BuChE than the positive control donepezil (IC_50_ = 6.21 µM). The most active compound, **65h**, also presented selectivity for this enzyme. Docking studies showed strong affinity for the active site of BuChE. Except for **65a**, all compounds were shown to be non-toxic for BV2 cells at 50 µM. Except for **66d**, all compounds were found to inhibit the production of nitric oxide (NO) in LPS-induced BV2 cells (1.04 < IC_50_ < 48.22 µM), the activity of compound **65a** (IC_50_ = 1.04 µM) being eight-fold higher than the positive control resveratrol (IC_50_ = 7.86 µM). It was found that the presence of substituents at the *meta* position of the benzene ring linked to the triazole moiety is more beneficial for activity than *ortho*- and *para*-substitution. Further studies showed that the compound **65h** exerts an anti-inflammatory effect through the inhibition of the expression of phosphorylated P65 protein, thus blocking the NF-κB signaling pathway. This compound also showed excellent inhibition of self-induced Aβ aggregation and resistance to H_2_O_2_-induced or Aβ-induced SH-SY5Y cytotoxicity. Compound **65h** was also revealed to have low hepatotoxicity, selective chelation with biometal ion Cu^2+^, and proper BBB permeability. Moreover, in vivo assays showed that **65h** improved the cognitive impairment in the scopolamine-induced AD mice model [110].

Considering the potential of tacrine, which was used for the treatment of AD, and aiming to develop new compounds using this moiety in combination with chalcones, also known for their neuroprotective potential, Rani et al. (2021) reported the synthesis of a series of 1,2,3-triazole-linked chalcone- and ferrocenylchalcone-tacrine conjugates and their anti-AChE activity [112]. The Click Chemistry step from corresponding chalcone azides and propargylated tacrine achieved the desired compounds in 64–78% yield. A total of 28 compounds (**69a**–**69w** and **70a**–**70e**, Figure 25) showed in vitro AChE inhibition (0.259 < IC_50_ <1.015 µM). Compounds **69i**, **69h** and **70e** (IC_50_ = 0.259, 0.372 and 0.327 µM, respectively) were found to be more active than the positive control tacrine (IC_50_ = 0.375 µM) and showed no acute toxicity. These three compounds were further screened against BuChE. Only compound **69i** showed above 50% inhibition at 10 μM, with an IC_50_ value of 5.328 µM, which suggests that these hybrids are selective inhibitors of AChE. The SAR analysis suggests that the presence of EWG on chalcone scaffold is more beneficial for activity than EDG, and the replacement of fluorine to chlorine increases activity. The length of the alkyl chain between the two pharmacophores also affects the activity. The substitution of the phenyl group for the ferrocenyl group on chalcones **70a**–**70e** did not improve the activity, except for compound **70e**, with the longer alkyl chain. In vivo assays and biochemical analysis of oxidative stress factors showed that the most promising compounds induce a substantial reversal of scopolamine-induced oxidative stress. Molecular docking studies of compounds **69h** and **69i** in the active site of AChE confirm the interaction of these compounds with residues of this enzyme [112].

### 4.2. Anti-Inflammatory Activity

**Figure 26:**
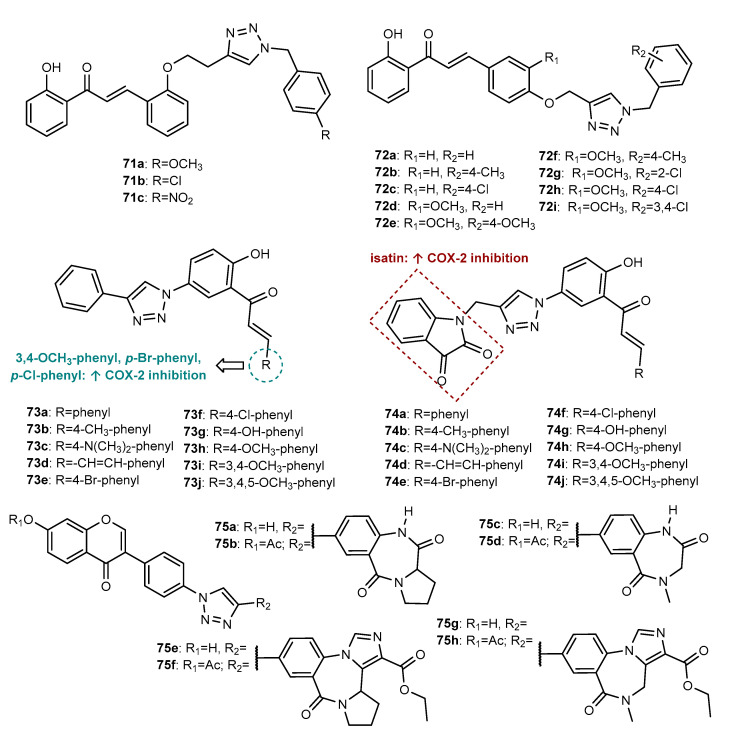
Flavonoid hybrids **71**–**75** with anti-inflammatory activity.

Considering the anti-inflammatory properties of chalcones and triazoles, and in order to obtain new inhibitors of 15-lipoxigenase (15-LOX), chalcone hybrids **71a**–**71c** and **72a**–**72i** with a triazole ring on the *ortho* position and *para* position of the B ring of the chalcone scaffold, respectively, (Figure 26) were synthesized [113]. Hybrids were obtained reacting *O*-propargyl-chalcones and benzyl halides by CuAAC reaction, giving rise to final products with 61–76% yield. The tested compounds showed moderate-to-weak 15-LOX inhibitory activity (12.2–40.3% inhibitory activity at 8 µM). Hybrid **72i**, with 40.3% of 15-LOX inhibition, presented the highest activity, followed by **72h** (35.1%).

Two series of 1,2,3-triazole-linked chalcone hybrids bearing a phenyl (**73a**–**73j**) or isatin (**74a**–**74j**) moiety (Figure 26) were synthesized by the CuAAC reaction between chalcone azides and phenyl acetylene or *N*-propargylisatin [114]. All compounds were screened in vitro for their inhibitory capacity against ovine COX-1 and human recombinant COX-2, having celecoxib and indomethacin as positive controls [114]. All compounds displayed higher inhibitory activity against COX-2 (0.037 < IC_50_ < 0.23 µM) than COX-1 (7.4 < IC_50_ < 13.7 µM) with COX-2 selectivity indexes ranging between 32.17 and 360.53. Among them, compounds **73e**–**73f**, **73i**, **74a**–**74c**, **74e** and **74h**–**74j** (0.037 < IC_50_ < 0.052 µM for COX-2) displayed an anti-inflammatory activity equivalent or even higher than celecoxib (IC_50_ = 0.045 µM). In general, substitution of benzene ring by isatin improved the COX-2 inhibitory activity. Additionally, all compounds showed significant in vitro 15-LOX inhibitory activity (1.41 < IC_50_ < 5.11 µM), higher than the positive control zileuton (IC_50_ = 15.6 µM). Among them, hybrids **73d**, **73f**, **73i**, **74c**, **74e** and **74h**–**74j** were found as potent dual inhibitors of COX-2 and 15-LOX. Using carrageenan-induced paw edema bioassay in rats, **73i**, **74c** and **74h** were identified as the most active compounds, showing between 109% and 116% of the in vivo anti-inflammatory activity of celecoxib. Moreover, docking studies on COX-2 and 15-LOX active sites ensured the binding affinity and selectivity [114]. Overall, compounds **74a**–**74j**, with an isatin moiety, showed an increased activity compared to **73a**–**73j**. In fact, isatin derivatives have been reported for their anti-inflammatory potential, namely, through COX-2 inhibition, which can explain these results. For the series of compounds **73a**–**73j**, the presence of 3,4-dimethoxy, *p*-bromo or *p*-chlorobenzene B ring was associated with an increase not only in the COX-2 inhibitory activity, but also in the selectivity towards COX-1 (Figure 26).

Mengheres et al. (2020) reported the synthesis and anti-inflammatory activity of eight isoflavone-benzodiazepine hybrids (**75a**–**75h**, Figure 26) obtained through the Click Chemistry approach from propargylated benzodiazepines and isoflavone azides [115]. All compounds were found to decrease in vitro the NO production on lipopolysaccharide (LPS)-stimulated BV2 mouse brain microglial cells, with 18.06–64.28% NO production at 20 µM, whereas natural isoflavones daidzein and formononetin showed 34.94% and 72.70% NO production, respectively, at the same concentration. Among them, compounds **75g** (19.99% NO production and 66.35% cell viability) and **75h** (19.88% NO production and 60.24% cell viability), having the same benzodiazepine scaffold, showed reasonable cell viability (>60%) and significant inhibition of NO production, being selected as the most promising compounds of this study [115].

Compounds **65a**–**65n**, **66a**–**66e**, **67a**–**67k** and **68a**–**68i** (Figure 25), reported previously in Section 4.1., were also screened for their anti-inflammatory activity using RAW264.7 cells stimulated with LPS [111]. The treatment of RAW264.7 cells with these hesperetin-1,2,3-triazole derivatives promoted a reduction in NO levels for all compounds (2.34 < IC_50_ < 19.87 µM), compound **68e** being the most active (IC_50_ = 2.34 µM). Moreover, almost all compounds showed a higher decrease in TNF-α, IL-1β and IL-6 production than the positive controls indomethacin and celecoxib on LPS-stimulated RAW264.7 cells at a concentration of 10 µM. The most promising compound, **68e**, also reduced the COX-2 expression in a concentration-dependent manner, negatively regulated the nuclear factor (NF)-kB signaling pathway and reduced the ROS production. In vivo studies showed that **68e** can reduce the histopathological changes in mice with acute liver injury (ALI) caused by intraperitoneal injection of CCl_4_, as well as the reduction in cytokines (TNF-α, IL-1β and IL-6) and liver enzymes aminotransferase (ALT) and aspartate aminotransferase (AST) expression [111].

### 4.3. Antioxidant Activity

The hesperetin hybrids **29a**–**29s** (Figure 12), reported in Section 2.3, were also screened for antioxidant activity using 2,2-diphenyl-1-picrylhydrazyl radical (DPPH^•^) and 2,2′-azino-bis(3-ethylbenzothiazoline-6-sulfonic acid) radical cation (ABTS^•+^) scavenging assays. All compounds were found to possess DPPH^•^ (30.75 < IC_50_ < 83.57 µM) and ABTS^•+^ (8.545 < IC_50_ < 39.356 µM) scavenging activity, comparable with the positive control ascorbic acid (DPPH^•^: IC_50_ = 12.72 µM, ABTS^•+^: IC_50_ = 5.0925 µM).

**Figure 27:**
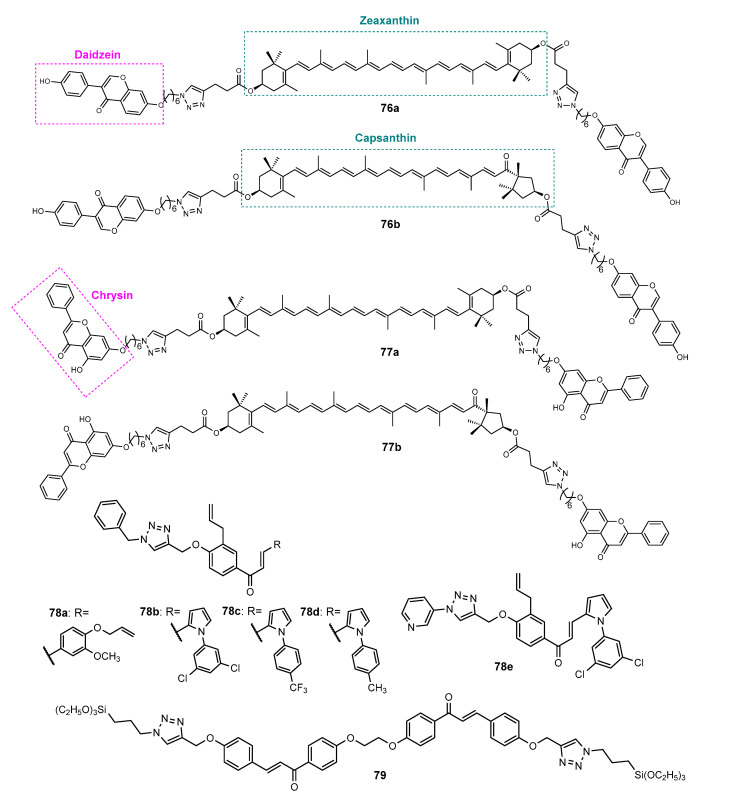
Flavonoid hybrids **76**–**79** with antioxidant activity.

Based on the antioxidant activity of the flavonoids and carotenoids, Linzembold et al. (2020) reported the conjugation of daidzein and chrysin with carotenoids (Figure 27). These hybrids were synthesized by Click Chemistry reaction in the presence of bis-triphenylphosphano-copper (I)-butyrate complex and DCM, giving rise to compounds **76a**–**76b** and **77a**–**77b** with 40–88% yield [116]. The trolox-equivalent antioxidant capacity (TEAC) of the synthetic conjugates was investigated towards ABTS^•+^ radical cation. Dimers of daidzein with zeaxanthin (**76a**) or capsanthin (**76b**) bearing a bistriazole ring showed a higher antioxidant effect than the respective carotenoids. While the dimers of chrysin with zeaxanthin (**77a**) showed higher antioxidant activity than the corresponding carotenoid, the dimer of chrysin with capsanthin (**77b**) did not improve the antioxidant activity. The synthesis and antioxidant activity of monotriazole flavonoid-carotenoid conjugates were also performed; however, all monotriazole derivatives revealed to be less active than the corresponding carotenoid.

Five chalcone hybrids (**78a**–**78e**, Figure 27) showed some antioxidant activity using DPPH radical scavenging assay [117]. The CuAAC reaction was performed in *O*-propargylated acetophenones used as building blocks, which were after used for the synthesis of chalcones **78a**–**78e** by Claisen–Schmidt condensation. All compounds were found to have moderate-to-low antioxidant activity (22.11 < IC_50_ < 50.88 µg/mL), when compared with the positive control vitamin C (IC_50_ = 12.64 µg/mL). At the highest concentration tested (1000 µg/mL), compounds displayed 71.24–91.48% DPPH^•^ scavenging activity (vitamin C: 98.78%).

The bis-chalcone-1,2,3-triazole-organosilane **79** (Figure 27) was found to display antioxidant activity in a total antioxidant activity (TAA) assay, through the ability to reduce the radical cation of ABTS, determined by the decoloration of ABTS^•+^ [118]. This compound, obtained by CuAAC in excellent yield in the presence of [CuBr(PPh_3_)_3_]/ TEA-THF system under inert atmosphere, showed a better antioxidant profile when compared with the acetylenic chalcone precursor and the positive control ascorbic acid [118].

### 4.4. Activity on Other Enzymes

Carbonic anhydrases (CA) are metalloenzymes involved in several physiological processes, including the maintenance of pH and bicarbonate homeostasis, respiration, bone metabolism and tumorigenesis. Different isoforms of CA are found in humans and differ not only in tissue localization but also in enzymatic efficiency. Several diseases are associated with a CA deregulation, such as glaucoma, hypertension, edema, epilepsy and cancer, so the development of CA inhibitors has been a strategy for the treatment of CA-related disorders [119,120]. 

**Figure 28:**
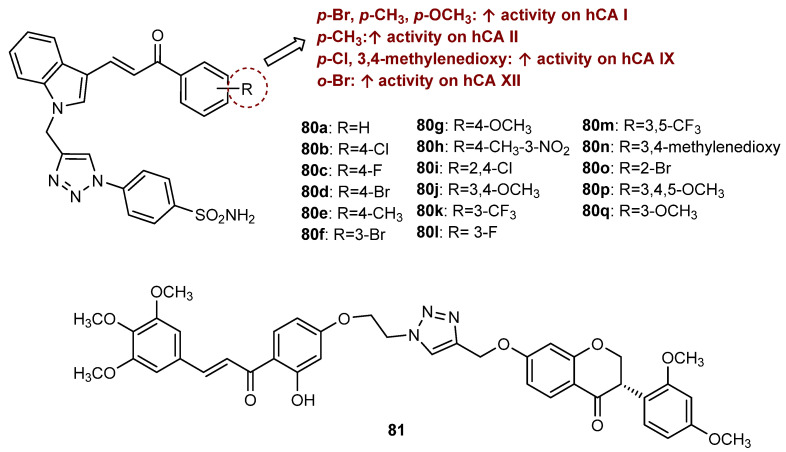
Flavonoid hybrids **80**–**81** with activity on other enzymes.

Considering the role of sulfonamide derivatives, including an indolyl sulfonamide, in CA inhibition, as well as the biological potential of the 1,2,3-triazole ring and chalcones, a series of 17 hybrids of indole chalcones with benzenesulfonamide through a 1,2,3-triazole linker (**80a**–**80q**, Figure 28) was synthesized by Singh et al. (2020) and evaluated for human carbonic anhydrases (hCA) inhibitory activity against the four physiologically and pharmacologically significant isoforms, the cytosolic isoforms hCA I and hCA II and the trans-membrane-tumor-associated isoforms, hCA IX and hCA XII [120]. CuAAC reaction occurred after the *N*-propargylation of the chalcone moiety, with benzenesulfonamide azide. All compounds were found to inhibit the cytosolic isoform hCAI, with Ki ranging between 18.8 nM and 5.5 µM. Among them, hybrids **80d** (Ki = 18.8 nM), **80e** (Ki = 50.4 nM) and **80q** (Ki = 38.3 nM) having a *p*-bromo, *p*-methyl and *m*-methoxybenzene A ring in the chalcone scaffold, respectively, were found as the most promising against hCA I isoform, with several times higher activity than the positive control acetazolamide (AAZ) (Ki = 250 nM). The hCA II isoform was also inhibited by all compounds with Ki from 36.2 nM to 9.7 µM, compound **80e** (Ki = 36.2 nM) with a *p*-methyl benzene ring being the most potent, comparable with AAZ (Ki = 12.1 nM). It was found that all compounds moderately inhibit the tumor-associated isoform hCA IX (69.3 nM < Ki < 1.4 µM), hybrids **80b** and **80n** being the most active, with Ki values of 73.3 and 85 nM, respectively. Inhibition of the tumor-associated isoform hCA XII was achieved in nanomolar range for all compounds, **80o**, **80m** and **80f** being the most active, with Ki values of 10 nM, 36.9 nM and 41.9 nM, respectively. Docking studies of the most active hCA I inhibitors **80d** and **80q** into the active pocket of hCA I suggest that both compounds were well accommodated in the active pocket of hCA I. Some SAR considerations concerning to the most active compounds for each hCA isoform are highlighted in Figure 28.

Compound **81** (Figure 28), a flavonoid dimer, which was obtained by Click Chemistry from previously propargylated isoflavanone and a chalcone azide, was shown to slightly inhibit serine proteases trypsin, chymotrypsin and elastase, by 39%, 74% and 53% at 100 µM, respectively [121].

Glutathione S-transferase (GST) is a phase II metabolic enzyme that plays a critical role in cellular detoxification against carcinogens, therapeutic drugs, and many products of oxidative metabolism. However, the overexpression of GST in cancer cells contributes to an increase in anticancer drug detoxification. Thus, the use of GST inhibitors can reverse tumor resistance through the suppression of GST activity and improvement of the chemotherapeutic drug sensitivity of tumor cells [122,123]. 

Beyond the inhibitory activity on AChE and BuChE enzymes, 1,2,3-triazole-linked chalcone derivatives **64a**–**64f** (Figure 25) were screened for their influence on GST inhibition, displaying inhibitory activity with IC_50_ = 10.86–15.10 µM and Ki = 9.82–13.22 µM [109]. The docking of the most active compound, **64d**, into the catalytic active site of GST showed that phenylene and triazole groups have the most important interactions for the inhibition of this enzyme.

### 4.5. Antidiabetic Activity

The study of the antidiabetic activity of flavonoid hybrids was limited to flavone and flavonol derivatives, which have been shown to act as α-glucosidase inhibitors and interfere with the glucose consumption of insulin-resistant (IR) HepG2 cells.

**Figure 29:**
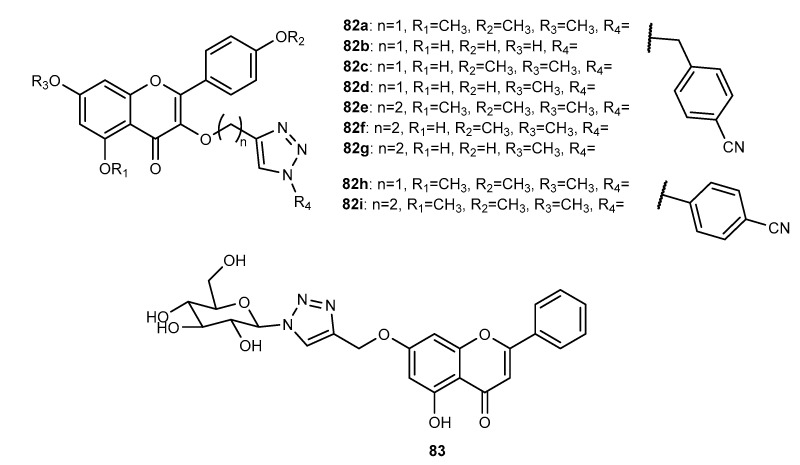
Flavonoid hybrids **82**–**83** with antidiabetic activity.

Kaempferol hybrids **82a**–**82i** (Figure 29) were synthesized by CuAAC reaction from *O*-propargylated kaempferol and aryl nitrile azides [124]. The antidiabetic activity of synthesized compounds was studied through the effect on the glucose consumption in IR HepG2 cells [124]. All compounds displayed higher activity (1.2 < EC_50_ < 155 nM) than standard drug metformin (EC_50_ = 258 nM). Particularly, **82f** (EC_50_ = 2.5 nM), **82g** (EC_50_ = 1.78 nM), **82h** (EC_50_ = 1.2 nM) and **82i** (EC_50_ = 2.1 nM) were found to be more potent than a flavonoid derivative identified previously as a lead compound by the same research group.

The 1,2,3-triazole-linked flavone-α-d-glucoside **83** (Figure 29) was obtained with 47% yield by Click Chemistry reaction between *O*-propargylated chrysin and α-d-glucosylazide in the presence of CuI, DIPEA and TBTA in acetonitrile, at room temperature [125]. This compound provided a strong inhibition of α-glucosidase (IC_50_ = 17 µM), 60-fold higher than the positive control acarbose (IC_50_ =1100 µM). Interestingly, it was found that the introduction of the glucosyl-triazole moiety improved the solubility and inhibition, when compared with flavone chrysin (IC_50_ = 77 µM).

### 4.6. Antifouling Activity

**Figure 30:**
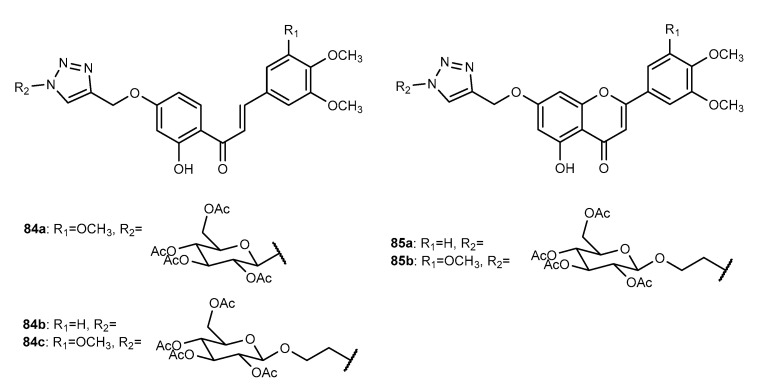
Flavonoid hybrids **84**–**85** with antifouling activity.

Marine biofouling is characterized by the colonization of macro and micro-organisms on submerged surfaces, which causes several material and economical concerns for the maritime industry. Moreover, environmental problems are associated with this phenomenon, due to the increase in fuel consumption and carbon dioxide emissions, and the spread of invasive species, which contributes to the loss of marine biodiversity. Since the biocides currently used in antifouling coatings have proved to be harmful, the search for effective and low toxic antifouling agents is an urgent request [38,126]. In an attempt to develop more environmentally friendly antifouling compounds, Pereira et al. (2021) synthesized a series of glycosylated chalcones and flavones conjugated through a triazole ring, through CuAAC reaction [127]. Compounds were screened for in vivo anti-settlement activity against the macrofouling organism *Mytilus galloprovincialis*. Five compounds, namely, chalcone derivatives **84a**–**84c** and flavone derivatives **85a**–**85b** (Figure 30) were found to display in vivo anti-settlement activity against the macrofouling organism *Mytilus galloprovincialis* (3.28 < EC_50_ < 53.90). Among them, compound **84a** (EC_50_ = 3.28 µM) showed the highest activity and the highest therapeutic ratio (LC_50_/EC_50_ > 60.98). Compound **84a** also displayed significant inhibitory activity of the biofilm-forming microalgae *Navicula* sp. (EC_50_ = 41.76 µM). Complementary ecotoxicity assay for the most active compound, **84a**, against the marine non-target organism *Artemia Salina* revealed that this compound is not toxic at concentrations of 25 and 50 µM, which makes it a potential environmentally friendly alternative to the antifoulants in use.

## 5. Conclusions

A large number of compounds (691) belonging to the 1,2,3-triazole-linked flavonoid hybrids reported since 2017 were highlighted in this review, largely concerning their synthesis and biological activities. Regarding the synthesis of the triazole ring, it was found that for the majority of studies, the Click Chemistry step was accomplished at room temperature using copper sulphate as a catalyst and sodium ascorbate as a reducing agent, with slight modifications on the solvent system, generally giving rise to the desired compounds in high yields. Nevertheless, the use of conventional heating or MW irradiation was also reported, with the advantage of the decrease in reaction time. Instead of copper sulphate, the use of other copper catalysts such as copper iodide or bromotris (triphenylphosphine) copper (I) was also described with good overall results. Interestingly, some reports focused the Click Chemistry step using cellulose-supported copper iodide nanoparticles as a reusable catalyst without loss of efficiency.

Among biological activities, antitumor and antimicrobial activities have been the most studied, mainly for chalcone derivatives. Some of derivatives of flavonoids with these activities were revealed to be more potent than parent compounds or even commercially available drugs, using in vitro assays. Specifically, among flavonoids with antitumor activity, a large number of compounds were revealed to be potent tumor growth inhibitors, hybrids of chalcone **16g** and flavone **33b** being the most potent, with IC_50_ values of nanomolar range (IC_50_ < 0.1 µM) in at least one tumor cell line. Moreover, flavonoid hybrids such as chalcone **12**, flavone **34** and isoflavone **42** showed higher growth inhibitory activity compared with the parent compounds and even some anticancer drugs. The investigation of the cellular and molecular mechanisms involved in the antiproliferative activity of some of these compounds confirmed the interference with apoptosis, tubulin polymerization and telomerase inhibition.

Considering flavonoids with antimicrobial activity, various chalcone hybrids, such as **49j**, **49n**, **50j**, **50p** and **50o**, showed comparable, or even higher, antibacterial activity to the reference drug ciprofloxacin, currently used for the treatment of microbial diseases. Moreover, the hybridization of chalcones with 4-aminoquinoline linked by the 1,2,3-triazole system through Click Chemistry allowed us to obtain compounds with promising antiplasmodial activity, highlighting compounds **47j**, **48b** and **48c**, which showed antiplasmodial activity against multidrug-resistant strains. These data suggest the potential of some flavonoid hybrids linked by the 1,2,3-triazole ring to circumvent microbiological diseases, especially those with multidrug resistance. Although several flavonoids have proven to have potent antitumor and antimicrobial activity in screening assays, the underlying molecular mechanism by which most of these hybrids suppress cancer or microorganism cell growth has been scarcely studied. Therefore, future exploitation of the mechanism of action of the most potent compounds may contribute to the discovery of innovative chemotherapeutic agents.

In addition to the antitumor and antimicrobial activity, several hybrids of flavonoids exhibited quite promising anti-inflammatory and antidiabetic activities. In fact, using in vitro and in vivo assays, chalcone-isatin hybrids **74c** and **74h** showed a significant and selective COX-2 inhibitory effect, which was equivalent to, or even higher than, celecoxib. Moreover, kaempferol hybrids **82a**–**82i** displayed a higher effect on the glucose consumption in IR HepG2 cells than standard drug metformin, suggesting their possible use in the discovery of new antidiabetic drug candidates.

Interestingly, some of these compounds also showed suitable pharmacokinetic and toxicity characteristics in in silico ADMET studies, reinforcing the fact that the flavonoid hybridization using 1,2,3-triazole as a linker may allow one not only to improve the potency, but also the pharmacokinetic profile of flavonoids. Nevertheless, in the future, complementary in vivo assays to clarify these data are mandatory.

## Data Availability

Not applicable.
